# Isolation and characterization of fMGyn-Pae01, a phiKZ*-*like jumbo phage infecting *Pseudomonas aeruginosa*

**DOI:** 10.1186/s12985-025-02679-w

**Published:** 2025-03-04

**Authors:** Kira Ranta, Mikael Skurnik, Saija Kiljunen

**Affiliations:** 1 HUS Diagnostic Center, Clinical Microbiology, University of Helsinki and Helsinki University Hospital, Helsinki, Finland; 2 Human Microbiome Research Program, Research Program Unit, Faculty of Medicine, University of Helsinki, Helsinki, Finland

**Keywords:** PhiKZ, Jumbo phage, *Pseudomonas aeruginosa*, Flagellum-dependent, Phage-binding receptor, Phage resistance, Phage therapy

## Abstract

**Background:**

*Pseudomonas aeruginosa* is an opportunistic pathogen that causes a wide variety of infections, and belongs to the group of ESKAPE pathogens that are the leading cause of healthcare-associated infections and have high level of antibiotic resistance. The treatment of infections caused by antibiotic-resistant *P. aeruginosa* is challenging, which makes it a common target for phage therapy. The successful utilization of phage therapy requires a collection of well characterized phages.

**Methods:**

Phage fMGyn-Pae01 was isolated from a commercial phage therapy cocktail. The phage morphology was studied by transmission electron microscopy and the host range was analyzed with a liquid culture method. The phage genome was sequenced and characterized, and the genome was compared to closest phage genomes. Phage resistant bacterial mutants were isolated and whole genome sequencing and motility, phage adsorption and biofilm formation assays were performed to the mutants and host bacterium.

**Results:**

The genomic analysis revealed that fMGyn-Pae01 is a lytic, phiKZ-like jumbo phage with genome size of 277.8 kb. No genes associated with lysogeny, bacterial virulence, or antibiotic resistance were identified. Phage fMGyn-Pae01 did not reduce biofilm formation of *P. aeruginosa*, suggesting that it may not be an optimal phage to be used in monophage therapy in conditions where biofilm formation is expected. Host range screening revealed that fMGyn-Pae01 has a wide host range among *P. aeruginosa* strains and its infection was not dependent on O-serotype. Whole genome sequencing of the host bacterium and phage resistant mutants revealed that the mutations had inactivated either a flagellar or *rpoN* gene, thereby preventing the biosynthesis of a functional flagellum. The lack of functional flagella was confirmed in motility assays. Additionally, fMGyn-Pae01 failed to adsorb on non-motile mutants indicating that the bacterial flagellum is the phage-binding receptor.

**Conclusion:**

fMGyn-Pae01 is a phiKZ-like jumbo phage infecting *P. aeruginosa*. fMGyn-Pae01 uses the flagellum as its phage-binding receptor, supporting earlier suggestions that flagellum might be utilized by phiKZ but differs from some other previous findings showing that phiKZ-like phages use the type-IV pili as the phage-binding receptor.

**Supplementary Information:**

The online version contains supplementary material available at 10.1186/s12985-025-02679-w.

## Background

*Pseudomonas aeruginosa* is a gram-negative, rod-shaped bacterium that causes opportunistic infections, including pneumonia, bacteremia, surgical wound infections and burn wound infections, mainly in immunocompromised patients [1–4]. *P. aeruginosa* is a member of the ESKAPE (*Enterococcus faecium*,* Staphylococcus aureus*,* Klebsiella pneumoniae*,* Acinetobacter baumannii*,* Pseudomonas aeruginosa* and *Enterobacter* spp) group of pathogens. The members of this group are the leading cause of healthcare-associated infection and are highly antibiotic resistant [1, 5]. *P. aeruginosa* can bind to various surfaces and form biofilms, which are usually more resistant to antibiotics than planktonic cells and tend to cause chronic infections [6–8]. Treatment of *P. aeruginosa* infections has become increasingly difficult due to its rising resistance towards antibiotics, which makes *P. aeruginosa* a common target for phage therapy [9, 10].

Bacteriophages (phages) are viruses that infect bacteria. Phages are categorized as lytic or temperate, with the latter group having both lytic and lysogenic life cycles. Phages can target any structural element on the bacterial cell surface [11–13] that they can then use as a binding receptor. After adsorption to the receptor, the phage ejects its genome into the bacterial cell thereby starting its life cycle. In the lytic cycle, phages immediately start to replicate their genomes within the bacterium, express their structural proteins and assemble the new virus particles that are released to the environment via cell lysis that kills the host bacterium. In the lysogenic life cycle, the phage genome integrates as a prophage into the host chromosomal DNA and replicates along with it until the prophage is induced to enter the lytic cycle [13, 14]. Phages are natural predators of bacteria and bacteria are able to develop resistance against phages to ensure their own survival. Mutations affecting the phage-binding receptor are most common and these mutations prevent the phage from binding to the bacterial surface [15, 16].

Phages usually have relatively small genomes under 200 kb. Phages with a genome size from 200 kb to 500 kb and > 500 kb are considered jumbo phages and mega phages, respectively [17, 18]. These phages usually have myovirus or siphovirus morphology and large capsids that allow the phages to accommodate their large genomes [19, 20]. Due to their large size, jumbo and mega phages may multiply slower and diffuse shorter distances on soft agar than smaller phages, thus generating tiny plaques that may remain undetected in phage enrichments. Hence, relatively little research has been carried out on them. Compared to smaller phages, jumbo phages carry more genes participating in genome replication and nucleotide metabolism [21, 22].

The first jumbo phage was reported in 1978 when phiKZ, a *P. aeruginosa* infecting myovirus with a large genome and virion size, was first characterized [23]. PhiKZ and other related phages have dsDNA genomes of approximately 280 kb that contain around 300 protein-encoding open reading frames (ORFs). They typically have head diameters of 105–140 nm and tails of 160–200 nm in length. PhiKZ-like phages have a special structure called the inner body (IB), a rod-like structure inside the capsid consisting of phage proteins, that has an important role during DNA packaging [24–26]. A distinct feature of phiKZ-like phages is the formation of a nucleus-like structure during the genome replication, which was first observed in *Pseudomonas chlororaphis* phage 201f2-1 [27]. The nuclear shell protein forming the structure is called chimallin (ChmA), the presence of which is a hallmark of the viral family *Chimalliviridae* [28].

In phage therapy, phages are used as alternatives to antibiotics in treating bacterial infections [29–31]. Phages used in phage therapy need to be strictly lytic and they should not carry genes encoding bacterial virulence-, toxicity- or antibiotic resistance-associated proteins [32, 33]. Temperate phages should be avoided, as phage therapy relies on phages to destroy the pathogenic bacteria and this feature is abolished with temperate phages turning to lysogenic growth cycle. In addition, prophages can potentially increase bacterial virulence [34].

When testing the sensitivity of Finnish clinical bacterial strains to commercial phage cocktails from Russia and Georgia (unpublished data), we found that one cocktail was effective against a clinical *P. aeruginosa* strain to which we had no phages. In this study we isolated and characterized this *P. aeruginosa* infecting phage, fMGyn-Pae01, from the cocktail. In addition, we demonstrated that phage fMGyn-Pae01 uses the *P. aeruginosa* flagellum as the phage-binding receptor.

## Materials and methods

### Bacterial strains, phage cocktail and phage isolation

All bacterial strains used in this work are presented in the Supplementary Table S1. Lysogeny Broth (LB) was used as growth medium. LB agar on 9 cm Petri dishes included additionally 1.5% agar and LB soft agar medium included additionally 0.3% agar. All bacteria and phage incubations were carried out at 37 °C [35].

fMGyn-Pae01 was isolated in 2019 from a commercial phage cocktail (MicroMir, Phagogyn, production date: September 02, 2016, expiration date: September 2018). A clinical *P. aeruginosa* strain (S6728) was used as the host strain (Supplementary Table S1). The phage cocktail was serially diluted to 10^− 3^ in LB and 50 µl of each dilution was incubated with the host strain overnight using the double-layer agar method. The host strain was grown in LB until the logarithmic phase was reached and the volume of bacteria mixed with 3 ml of soft agar was calculated from equation µl = 45 ÷ A_600_, where A_600_ was measured with Laxco DSM Cell Density Meter. This allowed formation of individual plaques on soft agar from which the phage was isolated using three rounds of plaque purification [35]. Phage titers in lysates were determined as plaque forming units (PFU)/ml based on dilution factor and number of plaques counted on plates.

fMGyn-Pae01 lysates were prepared using both semiconfluent plates and liquid cultures. To collect the phages from semiconfluent soft agar, 3 ml of SMG-buffer (100 mM NaCl, 10mM MgSO4, 50 mM Tris-HCl, pH 7.5, 0.01% (w/v) gelatin) was added to plates and incubated for 1–2 h at room temperature. The soft agar released to SMG buffer was collected and for each 3 ml of the obtained lysate 200 µl of chloroform was added and incubated at room temperature for 20 min by gently turning the tube up and down. The lysate was centrifuged at 3000 × *g* for 10 min or until supernatant was clear. The supernatant was filtered through a 0.2 μm filter. Sucrose was added to 8%. For lysates prepared from liquid cultures, 20 µl phage suspension and 200 µl of overnight culture of host bacteria were added to 5 ml LB and incubated at 37 °C with vigorous agitation for 5 h or until lysis occurred. The lysate was then treated with chloroform, centrifuged, filtered and sucrose was added as mentioned above. Phage titers of 2 × 10^11^ PFU/ml and 6 × 10^9^ PFU/ml were typically obtained with semiconfluent plates and liquid culture, respectively. Filtered phage lysate in LB was used for the assays unless otherwise stated.

### Electron microscopy

For electron microscopy, phage fMGyn-Pae01 was purified with ultrafiltration (Sartorius Vivaspin 300,000 MWCO ultrafiltration tubes) as described earlier [36] and LB was changed to 0.1 M ammonium acetate, pH 7, using the same column. The obtained phage preparation contained 9.4 × 10^10^ PFU/ml. A 3 µl aliquot of the phage preparation was transferred to a carbon-coated copper grid and allowed to absorb for one minute. The grid was stained with 2% uranyl acetate for 30 s. The sample was then examined with a transmission electron microscope (JEOL JEM-1400, Tokyo, Japan) under 80 kV at the Electron Microscopy Unit (Institute of Biotechnology, University of Helsinki, Helsinki, Finland). Pictures were taken using Gatan Orius SC 1000B bottom-mounted Charged Coupled Device (CCD)-camera (Gatan Inc., Pleasanton, CA, USA). Ten virions with contracted, and ten with non-contracted tails, were measured to determine the size and morphology of fMGyn-Pae01. ImageJ (release 1.52o) [37] was used for the measurements.

### DNA isolation and genome analysis

Phage DNA was isolated by phenol-chloroform extraction [35] and sequenced by Illumina HiSeq (2 ×x 150 bp) at Eurofins GATC Biotech. For *de novo* assembly of the genome, a 100,000-read subset was made from the original reads using Chipster [38] at IT Center for Science, Finland (CSC) and used for assembly with A5-miseq [39] (version 0.7.5a-r405) integrated pipeline for the *de novo* assembly of microbial genomes. PhageTerm [40] (version May 14, 2021) was used to predict the genome termini and the packaging method of the phage. The assembly was verified by mapping all the 19,153,760 original reads against the final genomic sequence with Geneious Prime^®^ (release 2022.1.1). Preliminary annotation was carried out with Prokka [41] (- v1.14.5 at KBase) and the final annotation was performed manually with BLASTp [42] (versions BLAST + 2.13.0 and BLAST + 2.14.0) and HHpred [43] (version 57c8707149031cc9f8edceba362c71a3762bdbf8). The genome was managed with Geneious Prime^®^ (release 2023.2.1) (https://www.geneious.com). The genome was screened for tRNA genes (tRNAscan-SE version 2.0 [44], Aragorn version v1.2.38 [45]), virulence genes (VirulenceFinder version 2.0 [46]) and antibiotic resistance genes (ResFinder version 3.1 [47]). All the bioinformatic tools were applied using standard parameters.

The annotated genomic sequence of fMGyn-Pae01 was deposited in NCBI GenBank under the accession number OR228460.1.

26 closest relatives of fMGyn-Pae01 were identified with Nucleotide BLAST analysis and used for construction of a phylogeny tree and a genome similarity heatmap. Phylogeny analysis was conducted with the VICTOR Virus Classification and Tree Building Online Resource [48] (https://victor.dsmz.de, accessed 21 February 2024) using the Genome-BLAST Distance Phylogeny (GBDP) method [49] under settings recommended for prokaryotic viruses [48]. The resulting intergenomic distances were used to infer a balanced minimum evolution tree with branch support via FASTME including Subtree Pruning and Regrafting (SPR) postprocessing [50] for the formula D0. Branch support was inferred from 100 pseudo-bootstrap replicates each. Trees were rooted at the midpoint [51] and visualized with ggtree [52]. Taxon boundaries at the species, genus and family level were estimated with the OPTSIL program [53], the recommended clustering thresholds [49] and an F value (fraction of links required for cluster fusion) of 0.5 [54]. The phylogenomic tree results as well as suggestions for the classification at the species, genus and family level were all obtained directly from VICTOR and do not necessarily reflect actual ICTV taxonomy. A genome similarity heatmap was generated using the VIRIDIC program (version v1.1) [55]. To create a three-phage genome-wide comparison, GFF3-files were downloaded from GenBank for phages fMGyn-Pae01 (OR228460.1), phiKZ (NC_004629.1) and OMKO1 (ON631220.1) and converted to BED-files in Galaxy Europe web platform (UseGalaxy.eu, Galaxy Version 1.0.1) [56]. The FASTA-files and BED-files were used to create an alignment with DiGAlign [57] (version 2.0). The start position of OMKO1 genome was adjusted to that of fMGyn-Pae01 and phiKZ for illustration purposes. The presence of 21 ORFs belonging to *Chimalliviridae* core genome and being present only in phages having chimallin [28] was verified with BLASTp (version + 2.16.0).

### Host range assay

fMGyn-Pae01 host range was tested on 142 bacterial strains, including 101 *P. aeruginosa*, 4 *Pseudomonas putida*, 10 *Escherichia coli*, 10 *Klebsiella pneumoniae*, 10 *Salmonella typhimurium* and 7 *Proteus mirabilis* strains (Supplementary Table S1). The host range screening was performed with a liquid culture method as previously described with small alterations [58]. Briefly, bacterial overnight cultures were diluted 1:500 in LB except for *P. aeruginosa* and *P. putida* that were diluted 1:40 in LB. 10 µl of phage lysate (1 × 10^9^ PFU/ml) and 190 µl diluted bacterial suspension were pipetted into the Honeycomb2-plate (Growth Curves AB Ltd) wells, corresponding to MOI (multiplicity of infection) of approximately 0.4 for *P. aeruginosa* and *P. putida* and approximately 3 for the rest of the strains. A negative control containing bacteria but no phage, a positive control with *P. aeruginosa* S6728 host strain and phage, and a blank control containing only LB were included to each plate. The samples were analyzed as duplicates. The assay was performed with Bioscreen C analyzer (Growth Curves AB Ltd, Finland) absorbance plate reader. The results were analyzed as previously described at the 10 h timepoint [58]. The blank was subtracted from all samples and the mean was calculated from the two parallel samples. The phage was considered to efficiently infect a given bacterial strain if the absorbance of the culture containing phage and bacteria was < 50% of the negative control containing bacteria but no phage. The phage was considered to infect with low efficiency if the culture absorbance was 50–70% of the negative control and to not infect a given bacterial strain if the culture absorbance was > 70% of the negative control.

### Isolation of phage resistant mutants

Phage resistant bacterial mutants were isolated by incubating 50 µl of host strain culture (OD_600_ ∼0.5) with 20 µl fMGyn-Pae01 (6 × 10^9^ PFU/ml) in 5 ml LB at 37 °C with vigorous agitation and continued after the culture lysis until new bacterial growth emerged. Samples from the culture were plated on LB agar and incubated at 37 °C overnight. Morphologically different single colonies were selected, and the bacterial isolates were purified from residual phage particles by making three sequential pure cultures of each bacterial strain. The resistance of the selected clones to fMGyn-Pae01 was confirmed with the same liquid culture method used in the host range screening assay.

### Bacterial DNA extraction, sequencing, and genome analysis

Bacterial DNA was extracted from *P. aeruginosa* host strain (S6728) and the phage resistant mutants (S6728-M5, S6728-M7, S6728-M8, S6728-M9 and S6728-M10) using the Invitrogen Jetflex Genomic purification kit. The whole genome sequencing (WGS) of the genomic DNA was performed at Novogene UK. The DNA libraries were sequenced using the Illumina paired-end 150 sequencing platform NovaSeq PE150. The trimmed and cleaned raw reads were obtained from Novogene and used for bioinformatics analyses.

Altogether approximately 12.6, 11.0, 14.2, 12.2, 11.4, and 11.4 million reads were received for S6728, S6728-M5, S6728-M7, S6728-M8, S6728-M9, and S6728-M10, respectively. Two million sequencing reads of the wild type strain S6728 were *de novo* -assembled using the Geneious assembler (Geneious Prime vs. 2023.2.1) resulting in 1342 contigs, 48 of which were longer than 3 kb, with a total sequence length of 6.91 mb. These contigs were used as reference sequences against which all the sequence reads of the phage resistant mutants were mapped using the Geneious assembler. All the SNPs, insertions and deletions against the 48 contigs were identified for each mutant. Those differences that were not common to all mutant strains were regarded as mutations occurring in genes that were the plausible causes for the phage resistance. The corresponding genes were identified from the *P. aeruginosa* strain 2021CK-01494 genome (GenBank acc no CP124669).

The raw reads of the *P. aeruginosa* host stain and of the fMGyn-Pae01 resistant mutants were deposited in NCBI Sequence Read Archive (SRA) under the BioProject accession number PRJNA1095914 (Supplementary Table S2).

### Motility assay

5 µl of overnight bacterial culture was inoculated into LB soft agar (0.4%) plates and incubated overnight. The assay was performed as triplicate for each bacterial strain. The diameter of each colony was measured from three different directions. The means and standard deviations were calculated with OriginPro 2021b (OriginLab Corporation, Northampton, MA, USA) using the three measurement results of all three parallel colonies. Paired-Sample t-Test was used to calculate standard deviations.

### Phage adsorption assay

Phage adsorption assay was performed to determine whether the mutations in fMGyn-Pae01 resistant strains affected phage-binding receptors. The host strain and phage resistant mutants were grown until OD_600_ ∼1.0. Then, 3.2 × 10^7^ PFU of fMGyn-Pae01 was mixed with 500 µl of bacteria, corresponding to MOI of approximately 0.1. The suspension was incubated at room temperature for 10 min after which the suspension was centrifuged at 13,000 × *g* for 3 min at room temperature, and the supernatant was recovered. The supernatant was treated with chloroform (300 µl, 15 min), centrifuged as previously, and the phage titer of unabsorbed phages in the supernatant was determined. The titrations were performed in triplicates. LB was used as an adsorption-free control without bacteria.

### Biofilm assay

Formation of biofilm was tested for S6728 and fMGyn-Pae01 resistant mutants with and without phage fMGyn-Pae01. Five ml of overnight bacterial culture in Tryptic Soy Broth (TSB, VWR Chemicals) was diluted 1:100 in TSB with 25% bovine plasma (Biowest). Then, 160 µl aliquots of this diluted bacterial suspension were pipetted into 96-wellplate wells. To the wells in which the ability of phages to prevent biofilm formation was tested, 10 µl of 1 × 10^8^ PFU/ml fMGyn-Pae01 was added before incubation. The assay was performed with six parallel samples. Negative controls without bacteria were also included. The plate was covered with parafilm and a lid, and incubated at 37 °C at 30–40 rpm for 48 h. After incubation, the liquid was carefully removed and 50 µl of 0.3% crystal violet was pipetted into the wells and incubated for 10 min. The excess crystal violet was removed, and the wells were washed four times with 200 µl H_2_O or until no more crystal violet was present in the wash. 200 µl of 95% EtOH was pipetted into the wells and incubated for 5 min. 1:10 dilutions in 95% EtOH were then prepared from the samples and 200 µl of each dilution was pipetted in triplicate to a fresh 96-well plate. The absorbance was measured at 540 nm using Hidex Sense Microplate Reader (Hidex, Turku, Finland).

## Results and discussion

### Phage isolation and morphology

**Fig. 1:**
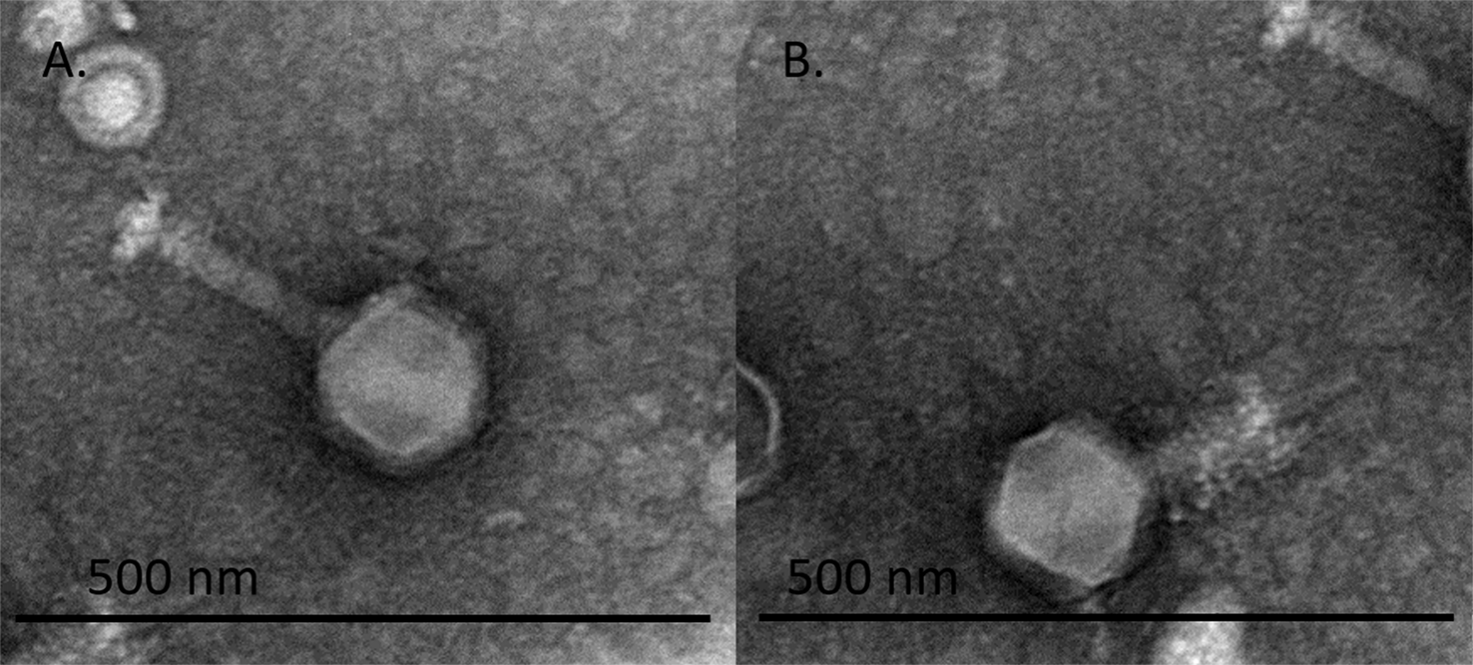
Transmission electron micrograph of fMGyn-Pae01. A purified phage sample was stained with 2% uranyl acetate and examined with a transmission electron microscope. Virions with non-contracted (**A**) and contracted (**B**) tails are shown. The morphology and the size of the virion was characteristic of myoviruses and jumbo phages

fMGyn-Pae01 was isolated from a commercial phage cocktail. Based on electron microscopy analysis phage fMGyn-Pae01 showed an icosahedral head and a contractile tail (Fig. 1). The measurements of a contracted and non-contracted fMGyn-Pae01 virion are presented in Table 1. The electron microscopy findings revealed that fMGyn-Pae01 had myovirus morphology with dimensions characteristic of jumbo phages [59].

**Table 1:** Contracted and non-contracted virions were measured to determine the dimensions (nm) of the fMGyn-Pae01 virion

Tail status	Head to tail	Tail sheath length (w/o baseplate and neck)	Tail sheath width	Head length	Head width
Non-contracted	298.91 (SD 7.56)	146.42 (SD 14.72)	31.87 (SD 1.72)	122.75 (SD 7.64)	123.64 (SD 6.10)
Contracted	226.42 (SD 10.97) (w/o tail tube)	74.50 (SD 6.24)	38.12 (SD 2.97)	122.76 (SD 7.03)	120.86 (SD 4.92)

### fMGyn-Pae01 is a phiKZ-like Jumbo phage

*De novo* assembly of fMGyn-Pae01 Illumina reads resulted to a single contiq of 277,803 bp genome encoding 358 predicted genes. The read coverage was 21.14 ×, and verification of the assembly by mapping all original reads to the assembled genome with approx. 7,900 read coverage did not reveal any mistakes in the sequence. Due to the large genome size, fMGyn-Pae01 is considered a jumbo phage. PhageTerm analysis did not identify distinct genome termini or packaging system. The preliminary analysis showed that fMGyn-Pae01 was similar to phiKZ, therefore, the reference genome of PhiKZ (GenBank acc no NC004629) was used to identify the genome start point. Genome CG-content was 36.9%. While 32 of the genes were predicted to code virion structural proteins, another 32 gene products were predicted to have functions in replication, recombination, repair, translation and transcription. Finally, 294 gene products were annotated as hypothetical proteins with unknown function. tRNAscan identified 7 tRNAs (Thr, Asn, Asn, Asp, Met, Leu, Pro) located between bp 265,166–270,956 of the genome.

VirulenceFinder and ResFinder did not identify any known gene products similar to virulence-, toxicity- or antibiotic resistance-associated proteins. Additionally, no genes encoding lysogeny-associated proteins were identified and therefore fMGyn-Pae01 meets the current safety requirements for therapeutic phages and is hence considered suitable for phage therapy. This result was expected since the phage was isolated from a product intended for phage therapy.

**Fig. 2:**
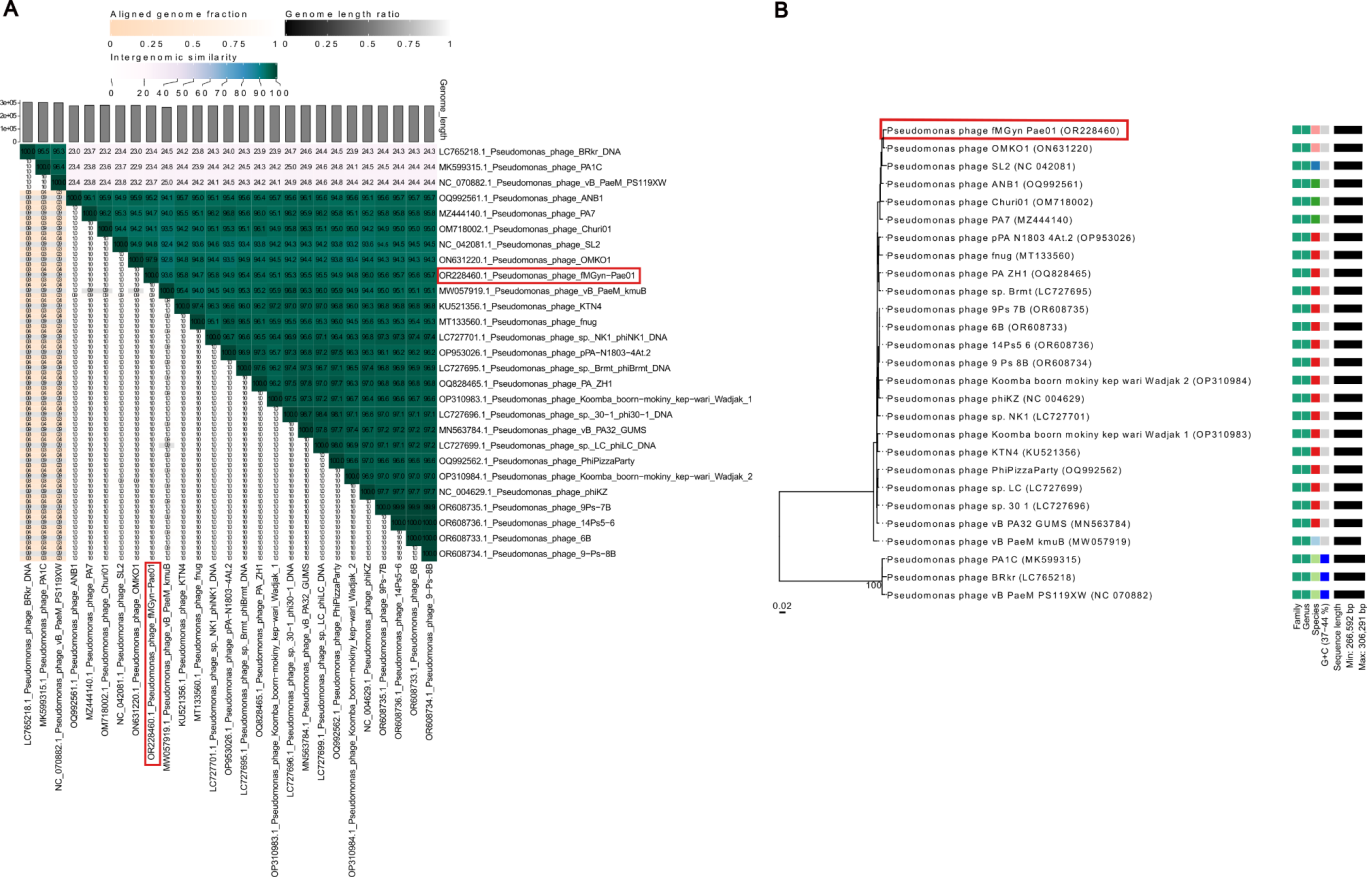
Heat map and phylogeny tree of fMGyn-Pae01 and 26 closest relatives based on nucleotide BLAST. (**A**) VIRIDIC heat map based on intergenomic similarities between fMGyn-Pae01 and closest relatives. (**B**) Phylogenetic tree using the distance formula D0, generated by VICTOR. fMGyn-Pae01 indicted with red box

A comparison of fMGyn-Pae01 genome to 26 closest phage genomes using VIRIDIC showed that the sequence identity was over 93% for the 23 closest genomes (Fig. 2A). The phylogeny tree generated by VICTOR (distance formula D0) showed that phages with the highest sequence identity based on VIRIDIC analysis, clustered together (Fig. 2B). All 26 closest phage genomes belong to *Pseudomonas* -specific jumbo phages that belong to order *Caudoviricetes*, of which the 23 closest ones cluster together. The closest relatives of fMGyn-Pae01 have been classified to family *Chimalliviridae* and genus *Phikzvirus* that contains three lytic *Pseudomonas* jumbo phages [60–62].

Prichard et al. (2023) identified 72 genes forming a core genome in chimallin-encoding phages, 21 out of which can only be found in phages encoding the chimallin protein. They suggested that this set of genes is likely to encode the proteins that form the phage nucleus, even though chimallin is the only protein whose function is currently known [28]. Out of these 21 genes, 20 are found in the phiKZ genome. When comparing these phiKZ gene products to fMGyn-Pae01, all 20 were found to be present in fMGyn-Pae01 with at least 95% amino acid identity to their phiKZ homologs (Table S3). It seems thus very likely that fMGyn-Pae01 forms the phage nucleus during its replication and should be considered as a member of *Chimalliviridae* family.

**Fig. 3:**
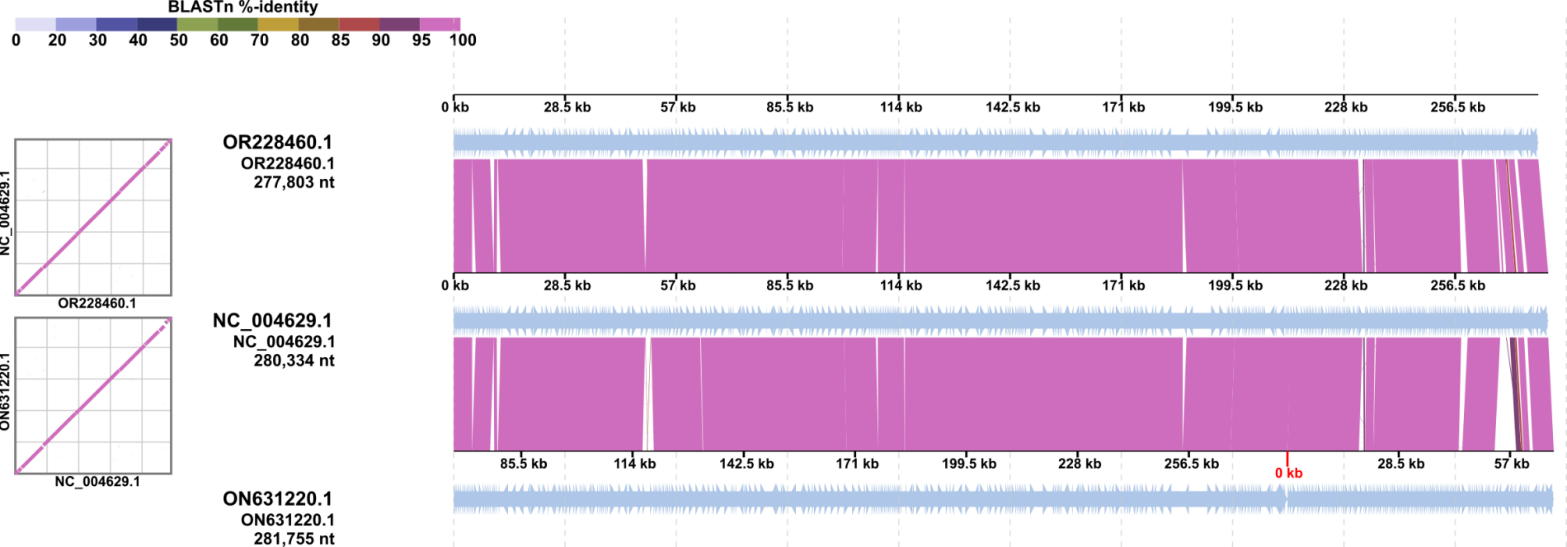
Whole-genome alignment of fMGyn-Pae01 (OR228460.1), phiKZ (NC_004629.1) and OMKO1 (ON631220.1). The alignment was performed with DiGAlign [57]. The start-site of OMKO1 genome was adjusted to fMGyn-Pae01 and phiKZ for illustration purposes, and the original start-site in the GenBank sequence is indicated in red

To carry out more detailed genomic comparison to closely related phages, a three-phage alignment between fMGyn-Pae01 (OR228460.1), phiKZ (NC_004629.1) and OMKO1 (ON631220.1) was performed with DiGAling. The three genomes align for their whole lengths (Fig. 3), the main differences being small insertions / deletions in genes coding for hypothetical proteins (not shown). The overall genomic identities in BLASTN between fMGyn-Pae01 and phiKZ and OMKO1 were 98.47% and 99.49%, respectively, indicating that the three phages should be regarded as strains of a single species.

### fMGyn-Pae01 has a wide host range

To determine the host range of fMGyn-Pae01, the infectivity of the phage was tested against 142 bacterial strains. Of the 101 *P. aeruginosa* strains used in the host range assay, fMGyn-Pae01 infected efficiently 43 strains (43%) and with low efficiency another 7 strains (7%) (Supplementary Table S1). Serotyping of *P. aeruginosa* is based on differences among the lipopolysaccharide (LPS) O-antigen side chains. The *P. aeruginosa* strains used in the assay included the 20 IATS (International Antigen Typing Scheme) O-serotype reference strains and four defined LPS mutants. fMGyn-Pae01 infected efficiently 8 of the 20 IATS-strains and one strain with low efficiency. It also infected the LPS-mutants missing either the O-specific (OSA) or the common polysaccharide (CPA) antigen (strains 6725 and 6726, Supplementary Table S1), but not the *rmlC* and *wbpL* mutants that miss both OSA and CPA. These findings indicate that fMGyn-Pae01 has a broad host range, and that the phage sensitivity is not dependent of the O-serotype of the strain. A broad host range is desirable for phages considered for phage therapy. fMGyn-Pae01 did not infect any of the other bacterial species except *P. aeruginosa* used in the host range assay.

### fMGyn-Pae01 utilizes the bacterial flagellum as its receptor

As fMGyn-Pae01 infection was not dependent on O-serotype, to identify the phage receptor, five fMGyn-Pae01 resistant mutants (S6728-M5, -M7, -M8, -M9, -M10) were isolated and subjected to WGS. All mutant strains had genomic changes that differed from the wild type strain S6728 (Table 2). Mutants S6728-M5 and S6728-M7 both had a 25 bp deletion affecting the flagellar *flgK*-gene and were genetically identical. S6728-M9 and S6728-M10 also had mutations affecting the flagellum biosynthesis. S6728-M9 had a 111 bp deletion affecting the *fliC* gene and S6728-M10 had a 3 bp substitution affecting the stop codon of *fliE.* S6728-M8 had a single amino acid substitution in the gene encoding RpoN (sigma factor 54) that has a vital role in regulating traits like nitrogen metabolism, motility and biofilm formation [63, 64].

**Table 2:** Mutations leading to fMGyn-Pae01 resistance

Mutant	Gene	mutation	Explanation	Reference
S6728-M5	*flgK*	25 bp deletion	Hook-filament junction	Erhardt and Huges 2010 [83]
S6728-M7	*flgK*	25 bp deletion	Hook-filament junction	Erhardt and Huges 2010 [83]
S6728-M8	*rpoN*	R403W substitution (corresponds to R383 of RpoN in *Klebsiella pneumoniae*, see Xiao et al. 2009 [84])	Sigma factor RpoN. Regulates motility	Xiao et al. 2009 [84], Francke et al. 2011 [63]
S6728-M9	*fliC*	111 bp deletion	Flagellin	Erhardt and Huges 2010 [83]
S6728-M10	*fliE*	A 3 bp substitution changes the TGA stop codon of *fliE* to CTC. Extending the C-terminus by pentapeptide LAARG*	Flagellum rod anchor	Hendriksen et al. 2021 [85]

**Fig. 4:**
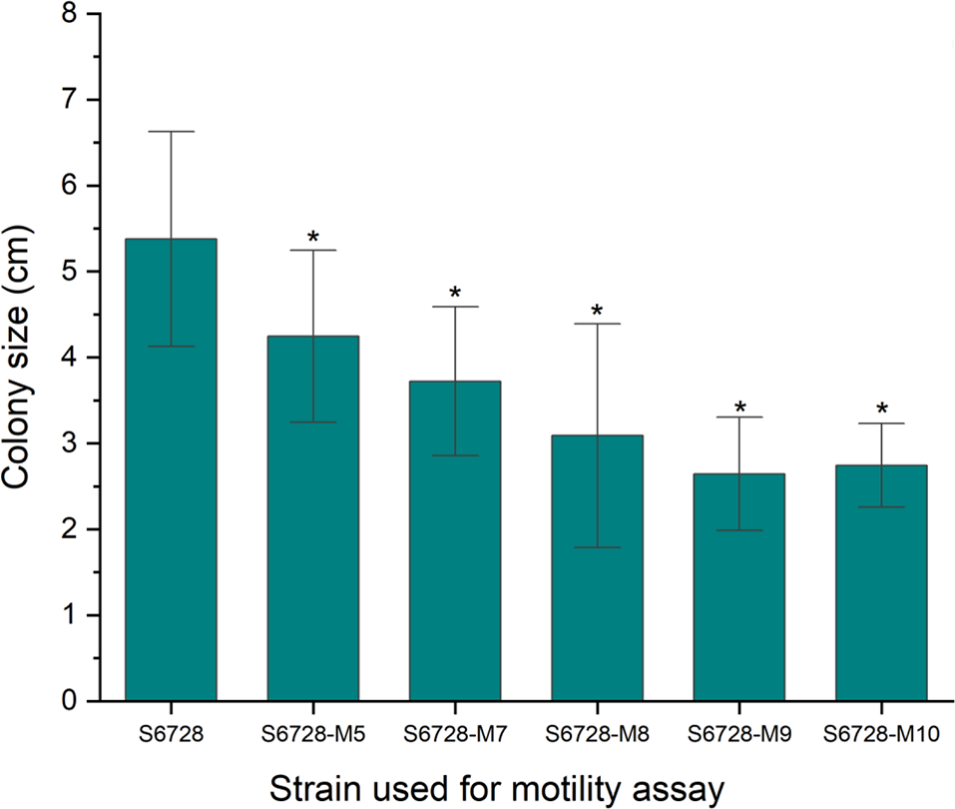
Motility results of host strain and phage resistant mutants. Shown are the diameters of the bacterial colonies after overnight incubation. All phage resistant mutants had decreased motility compared to host strain. Symbol * indicates that the values are significantly different (*p* < 0.05). Calculations and visualization were performed with OriginPro 2021b (OriginLab Corporation, Northampton, MA, USA). Paired-Sample t-Test was used to calculate standard deviations

The motility of the wild type strain and the fMGyn-Pae01 resistant mutants was measured on soft agar plates. All phage resistant mutants showed significantly decreased motility compared to the wild type strain (Fig. 4 and Supplementary Table S4). The results of the motility assay confirmed the WGS results indicating that mutations affecting flagellar biosynthesis prevented expression of functional flagellum and hence affected motility of the strains. The single amino acid substitution of RpoN also decreased motility. It is likely, that this substitution inhibits the binding of RpoN to the promoter elements and hence affects the transcription of the flagellar genes. RpoN is a positive regulator of flagellin gene expression and hence essential for the expression functional flagellae [63, 65].

**Fig. 5:**
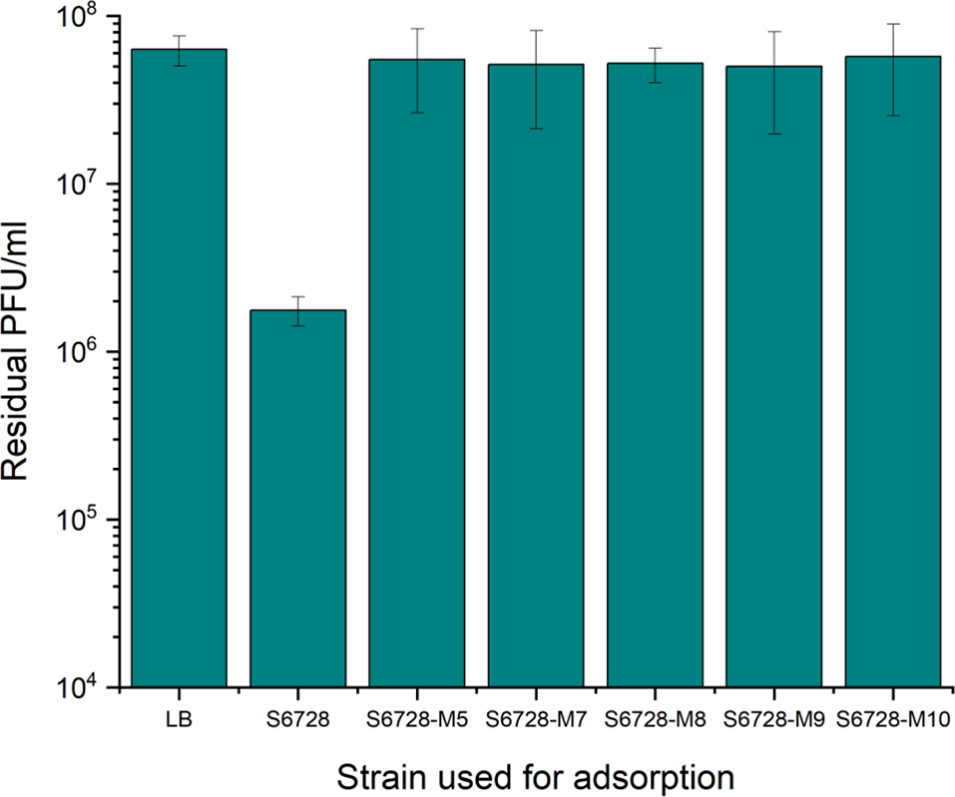
Adsorption of fMGyn-Pae01 to wild type *P. aeruginosa* strain and to phage resistant mutants. Shown are the titers of non-adsorbed phages in the supernatants after incubation with the bacterial strains. LB indicates an adsorption-free control without bacteria. fMGyn-Pae01 successfully adsorbed on host strain S6728 but not to the phage resistant mutants. Calculations and visualization were performed with OriginPro 2021b (OriginLab Corporation, Northampton, MA, USA)

Adsorption of fMGyn-Pae01 to host strain S6728 and phage resistant bacterial mutants S6728-M5, S6728-M7, S6728-M8, S6728-M9 and S6728-M10 was determined to clarify whether the mutations affected the phage receptors (Fig. 5). fMGyn-Pae01 successfully adsorbed to host strain but was not able to adsorb to any of the phage resistant strains, indicating that fMGyn-Pae01 uses the bacterial flagellum as phage receptor to initiate infection. This finding is supported by earlier studies showing that phiKZ-like phages have one distinct DNA injection site close to the cell pole, which has led to suggestion that these phages might use a polarly localized receptor such as flagellum as their receptor [66] and a preprint by Li et al. (2022) showing that flagellum is needed for phiKZ attachment [67]. On the other hand, some PhiKZ-like phages have previously been identified to be type-IV pili dependent [68] and the infection of OMKO1 was shown to be inhibited in a D*oprM* knockout mutant, which led to hypothesis that outer membrane porin M (OprM) may be the phage receptor [69]. However, even though OprM was shown to be needed for OMKO1 infection, the authors did not perform an adsorption assay to analyze if also phage adsorption was abolished in the D*oprM* strain. As tail fiber proteins of fMGyn-Pae01 and OMKO1 are 100% identical (not shown), it is likely that flagellum is the primary attachment receptor for OMKO1 as well. To date, there are only few published studies showing that jumbo phages or *P. aeruginosa* specific phages would bind to the bacterial flagellum. A review by Esteves and Scharf in 2022 [70] summarizes that flagellotropic phages infect both gram-negative and gram-positive bacteria. In their review, the only flagellotropic jumbo phages were PBS1 infecting *Bacillus subtilis* and FCbK infecting *Caulobacter crescentus.* Only one *P. aeruginosa* specific phage, phiCTX having a 35.6 kb genome, was identified to use the bacterial flagellum as its binding receptor [71–74]. Furthermore, at least one flagellotropic jumbo phage infecting *Klebsiella aerogenes* has been identified after the review by Esteves and Scharf was published [75]. In addition to type-IV pili and flagella, LPS and capsular polysaccharides has been identified as binding receptors used by jumbo phages [76–78].

Using flagellotropic phages in therapeutic phage cocktails can be advantageous in many ways: Motility is an important virulence factor for many bacteria, thus, the phage resistant mutants emerging during the therapy are likely to be less virulent. Second, flagellar motility is a very energy-consuming process, which directs flagellotropic phages to select for host cells having high fitness and providing optimal capacity for phage replication. Third, rotating flagellum covers a wide area, which improves the likelihood of the phage to find the bacterial cell [75]. Fourth, as all known bacterial flagellins share a certain conserved domain architecture [70, 79], one can hypothesize that binding to flagella may provide broader host range when compared to LPS/capsule -specific phages.

### fMGyn-Pae01 does not prevent biofilm formation

**Fig. 6:**
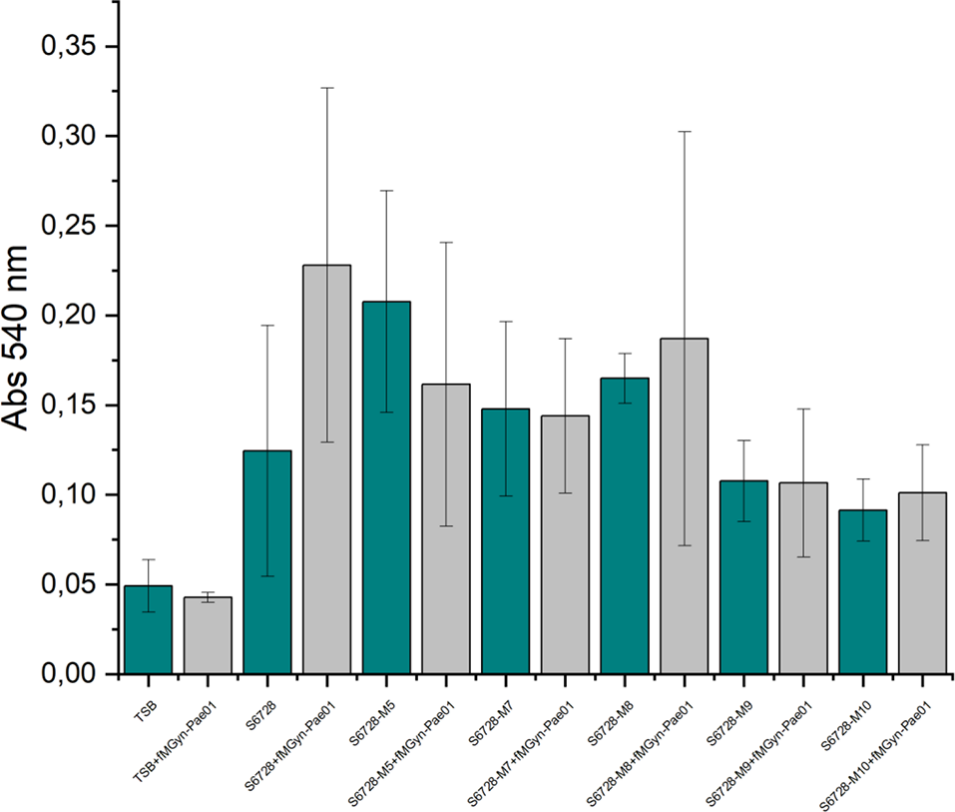
Biofilm assay results. Biofilm formation of wild type strain and phage resistant mutant strains was evaluated as well as and the ability of fMGyn-Pae01 to inhibit biofilm formation. The green bars show biofilm formation in the absence of fMGyn-Pae01, and grey bars show the results in the presence of fMGyn-Pae01. Calculations and visualization were performed with OriginPro 2021b (OriginLab Corporation, Northampton, MA, USA)

The ability to form biofilm was assessed for the wild type strain and the phage resistant mutants (Fig. 6). All bacterial strains formed biofilm to some degree. Interestingly, mutants S6728-M5, S6728-M7 and S6728-M8 formed slightly more, and mutants S6728-M9 and S6728-M10, formed equal or slightly less biofilm compared to wild type strain.

The potential of fMGyn-Pae01 to inhibit biofilm formation was also evaluated (Fig. 6). Previously, phages have been reported to inhibit biofilm formation presumably using polysaccharide depolymerases that degrade extracellular polymeric substances forming extracellular matrix protecting bacterial cells [80]. The results obtained in this study, however, showed that fMGyn-Pae01 did not have any inhibitory effect on biofilm formation. Interestingly, on the contrary, fMGyn-Pae01 increased slightly, but not significantly, biofilm formation in the wild type strain. In BLASTp analysis, the tail fiber protein identified in the genome of fMGyn-Pae01 (protein id WNV47846.1) had putative collagen-like conserved domains and no similarity to depolymerases, which is a logical finding for a phage using a filamentous protein as its receptor. Furthermore, no halo was detected around the phage plaques. All the findings thus support the conclusion that fMGyn-Pae01 does not have anti-biofilm activity. However, when analysing the results of the biofilm assay, one should keep in mind that some of the formed biofilms were poorly attached to the bottom of the well or were free-floating and slimy. This affected the staining and washing of the biofilms and thus could have affected the results obtained in the assay even though the assay was performed with six parallel samples. The technical challenges in the assay can be seen as a high margin of error, and not too definitive conclusions should be made based on these results.

RpoN has previously been identified to be necessary also for biofilm formation [63, 80, 81]. However, the results obtained from the biofilm assay in this study indicated that a fully functional RpoN is not needed for biofilm formation. Although the single amino acid substitution affecting RpoN in S6728-M8 affected the biosynthesis of functional flagellum, it did not inhibit the strain from producing biofilm.

## Conclusion

A lytic, phiKZ-like jumbo phage, fMGyn-Pae01, was isolated from a commercial phage cocktail. The isolated phage has a wide host range, although out of the bacterial panel tested in this work, it only infected strains belonging to *P. aeruginosa* species. The phage uses the bacterial flagellum as its phage-binding receptor, which supports earlier suggestions that flagellum might be utilized by phiKZ but differs from some other previous findings showing that phiKZ-like phages use the type-IV pili as the phage-binding receptor. No genes encoding bacterial virulence-, toxicity-, antibiotic resistance- or lysogeny-associated proteins were identified, confirming that fMGyn-Pae01 can be considered safe for phage therapy. On the other hand, fMGyn-Pae01 did not inhibit the biofilm formation of bacteria, which may reduce the therapeutic potential of fMGyn-Pae01 if used as a monophage in diseases where biofilms are considered high risk. However, the broad host range which does not depend on the O-serotype of the *P. aeruginosa* strain and the facts that fMGyn-Pae01 was isolated from a commercial phage therapy cocktail and the highly similar phage OMKO1 has been successfully used for phage therapy of a patient having aortic graft infection [82], support the conclusion that fMGyn-Pae01 is suitable for phage therapy.

## Electronic supplementary material

Below is the link to the electronic supplementary material.

Supplementary Material 1

## Data Availability

The annotated genomic sequence of fMGyn-Pae01 was deposited in NCBI GenBank under the accession number OR228460.1. The raw reads of the P. aeruginosa host stain and of the fMGyn-Pae01 resistant mutants were deposited in NCBI Sequence Read Archive (SRA) under the BioProject accession number PRJNA1095914.

## References

[1] Pendleton JN, Gorman SP, Gilmore BF. Clinical relevance of the ESKAPE pathogens. Expert Rev Anti Infect Ther. 2013;11:297–308. 10.1586/eri.13.12. 23458769 10.1586/eri.13.12

[2] Rello J, Borgatta B, Lisboa T. Risk factors for *Pseudomonas aeruginosa* pneumonia in the early twenty-first century. Intensive Care Med. 2013;39(12):2204–6. 10.1007/s00134-013-3046-1. 24146002 10.1007/s00134-013-3046-1

[3] Kang CI, Kim SH, Kim HB, Park SW, Choe YJ, Oh M, Kim EC, Choe KW. *Pseudomonas aeruginosa* bacteremia: risk factors for mortality and influence of delayed receipt of effective antimicrobial therapy on clinical outcome. Clin Infect Dis. 2003;37(6):745–51. 10.1086/377200. 12955633 10.1086/377200

[4] Turner KH, Everett J, Trivedi U, Rumbaugh KP, Whiteley M. Requirements for *Pseudomonas aeruginosa* acute burn and chronic surgical wound infection. PLoS Genet. 2014;10(7):e1004518. 10.1371/journal.pgen.1004518. 25057820 10.1371/journal.pgen.1004518 PMC4109851

[5] De Oliveira DMP, Forde BM, Kidd TJ, Harris PNA, Schembri MA, Beatson SA, Paterson DL, Walker MJ. Antimicrobial resistance in ESKAPE pathogens. Clin Microbiol Rev. 2020;33:e00181–19. 10.1128/CMR.00181-19. 32404435 10.1128/CMR.00181-19 PMC7227449

[6] Mah TF, Pitts B, Pellock B, Walker GC, Stewart PS, O’Toole GA. A genetic basis for *Pseudomonas aeruginosa* biofilm antibiotic resistance. Nature. 2003;426:306–10. 10.1038/nature02122. 14628055 10.1038/nature02122

[7] Costerton JW, Stewart PS, Greenberg EP. Bacterial biofilms: a common cause of persistent infections. Science. 1999;284:1318–22. 10.1126/science.284.5418.1318. 10334980 10.1126/science.284.5418.1318

[8] Stewart PS, Costerton JW. Antibiotic resistance of bacteria in biofilms. Lancet. 2001;358(9276):135–8. 10.1016/s0140-6736(01)05321-1. 11463434 10.1016/s0140-6736(01)05321-1

[9] Pang Z, Raudonis R, Glick BR, Lin TJ, Cheng Z. Antibiotic resistance in *Pseudomonas aeruginosa*: mechanisms and alternative therapeutic strategies. Biotechnol Adv. 2019;1:177–92. 10.1016/j.biotechadv.2018.11.013. 10.1016/j.biotechadv.2018.11.013 30500353

[10] Pires DP, Vilas Boas D, Sillankorva S, Azeredo J. Phage therapy: a step forward in the treatment of *Pseudomonas aeruginosa* infections. J Virol. 2015;89(15):7449–56. 10.1128/JVI.00385-15. 25972556 10.1128/JVI.00385-15 PMC4505681

[11] Gonzalez F, Helm RF, Broadway KM, Scharf BE. More than rotating flagella: lipopolysaccharide as a secondary receptor for flagellotropic phage 7-7-1. J Bacteriol. 2018;200:e00363–18. 10.1128/JB.00363-18. 30012730 10.1128/JB.00363-18 PMC6148473

[12] Fong K, Wong CWY, Wang S, Delaquis P. How broad is enough: the host range of bacteriophages and its impact on the agri-food sector. PHAGE. 2021;2:83–91. 10.1089/phage.2020.0036. 36148040 10.1089/phage.2020.0036 PMC9041489

[13] Campbell A. The future of bacteriophage biology. Nat Rev Genet. 2003;4(6):471–7. 10.1038/nrg1089. 12776216 10.1038/nrg1089 PMC7097159

[14] Cisek AA, Dąbrowska I, Gregorczyk KP, Wyżewski Z. Phage therapy in bacterial infections treatment: one hundred years after the discovery of bacteriophages. Curr Microbiol. 2017;74(2):277–83. 10.1007/s00284-016-1166-x. 27896482 10.1007/s00284-016-1166-x PMC5243869

[15] Hasan M, Ahn J. Evolutionary dynamics between phages and bacteria as a possible approach for designing effective phage therapies against antibiotic-resistant bacteria. Antibiot (Basel). 2022;11:915. 10.3390/antibiotics11070915. 10.3390/antibiotics11070915 PMC9311878 35884169

[16] Georjon H, Bernheim A. The highly diverse antiphage defence systems of bacteria. Nat Rev Microbiol. 2023;21:686–700. 10.1038/s41579-023-00934-x. 37460672 10.1038/s41579-023-00934-x

[17] Nazir A, Ali A, Qing H, Tong Y. Emerging aspects of Jumbo bacteriophages. Infect Drug Resist. 2021;14:5041–55. 10.2147/IDR.S330560. 34876823 10.2147/IDR.S330560 PMC8643167

[18] Devoto AE, Santini JM, Olm MR, Anantharaman K, Munk P, Tung J, Archie EA, Turnbaugh PJ, Seed KD, Blekhman R, Aarestrup FM, Thomas BC, Banfield JF. Megaphages infect *Prevotella* and variants are widespread in gut microbiomes. Nat Microbiol. 2019;4(4):693–700. 10.1038/s41564-018-0338-9. 30692672 10.1038/s41564-018-0338-9 PMC6784885

[19] Hua J, Huet A, Lopez CA, Toropova K, Pope WH, Duda RL, Hendrix RW, Conway JF. Capsids and genomes of jumbo-sized bacteriophages reveal the evolutionary reach of the HK97 fold. mBio. 2017;8(5). 10.1128/mbio.01579-17. 10.1128/mBio.01579-17 PMC5646251 29042498

[20] Iyer LM, Anantharaman V, Krishnan A, Burroughs AM, Aravind L. Jumbo phages: a comparative genomic overview of core functions and adaptions for biological conflicts. Viruses. 2021;13:63. 10.3390/v13010063. 33466489 10.3390/v13010063 PMC7824862

[21] De Smet J, Zimmermann M, Kogadeeva M, Ceyssens PJ, Vermaelen W, Blasdel B, Bin Jang H, Sauer U, Lavigne R. High coverage metabolomics analysis reveals phage-specific alterations to *Pseudomonas aeruginosa* physiology during infection. ISME J. 2016;10(8):1823–35. 10.1038/ismej.2016.3. 26882266 10.1038/ismej.2016.3 PMC5029163

[22] Yuan Y, Gao M. Jumbo bacteriophages: an overview. Front Microbiol. 2017;14:403. 10.3389/fmicb.2017.00403. 10.3389/fmicb.2017.00403 PMC5348500 28352259

[23] Krylov V, Bourkaltseva M, Pleteneva E, Shaburova O, Krylov S, Karaulov A, Zhavoronok S, Svitich O, Zverev V. Phage phiKZ-the first of giants. Viruses. 2021;13(2):149. 10.3390/v13020149. PMID: 33498475; PMCID: PMC7909554. 33498475 10.3390/v13020149 PMC7909554

[24] Nichiporenko A, Antonova D, Kurdyumova I, Khodorkovskii M, Yakunina MV. Assembly of PhiKZ bacteriophage inner body during infection. Biochem Biophys Res Commun. 2024;693:149372. 10.1016/j.bbrc.2023.149372. 38128246 10.1016/j.bbrc.2023.149372

[25] Sokolova OS, Shaburova OV, Pechnikova EV, Shaytan AK, Krylov SV, Kiselev NA, Krylov VN. Genome packaging in EL and Lin68, two giant phiKZ-like bacteriophages of *P. aeruginosa*. Virology. 2014;468(70):472–78. 10.1016/j.virol.2014.09.002 25254945 10.1016/j.virol.2014.09.002

[26] Krylov V, Dela Cruz D, Hertveldt K, Ackermann HW. φKZ-like viruses, a proposed new genus of myovirus bacteriophages. Arch Virol. 2007;152:1955–9. 10.1007/s00705-007-1037-7. 17680323 10.1007/s00705-007-1037-7

[27] Chaikeeratisak V, Nguyen K, Khanna K, Brilot AF, Erb ML, Coker JK, Vavilina A, Newton GL, Buschauer R, Pogliano K, Villa E, Agard DA, Pogliano J. Assembly of a nucleus-like structure during viral replication in bacteria. Science. 2017;355(6321):194–7. 10.1126/science.aal2130. 28082593 10.1126/science.aal2130 PMC6028185

[28] Prichard A, Lee J, Laughlin TG, Lee A, Thomas KP, Sy AE, Spencer T, Asavavimol A, Cafferata A, Cameron M, Chiu N, Davydov D, Desai I, Diaz G, Guereca M, Hearst K, Huang L, Jacobs E, Johnson A, Kahn S, Koch R, Martinez A, Norquist M, Pau T, Prasad G, Saam K, Sandhu M, Sarabia AJ, Schumaker S, Sonin A, Uyeno A, Zhao A, Corbett KD, Pogliano K, Meyer J, Grose JH, Villa E, Dutton R, Pogliano J. Identifying the core genome of the nucleus-forming bacteriophage family and characterization of *Erwinia* phage RAY. Cell Rep. 2023;42(5):112432. 10.1016/j.celrep.2023.112432. 37120812 10.1016/j.celrep.2023.112432 PMC10299810

[29] Rohde C, Resch G, Pirnay J-P, Blasdel BG, Debarbieux L, Gelman D, Górski A, Hazan R, Huys I, Kakabadze E, et al. Expert opinion on three phage therapy related topics: bacterial phage resistance, phage training and prophages in bacterial production strains. Viruses. 2018;10:178. 10.3390/v10040178. 29621199 10.3390/v10040178 PMC5923472

[30] Kortright KE, Chan BK, Koff JL, Turner PE. Phage therapy: a renewed approach to combat antibiotic-resistant bacteria. Cell Host Microbe. 2019;25:219–32. 10.1016/j.chom.2019.01.014. 30763536 10.1016/j.chom.2019.01.014

[31] Pirnay JP, Djebara S, Steurs G, Griselain J, Cochez C, De Soir S, Glonti T, Spiessens A, Vanden Berghe E, Green S, Soentjens P, Lavigne R, Merabishvili M, et al. Personalized bacteriophage therapy outcomes for 100 consecutive cases: a multicentre, multinational, retrospective observational study. Nat Microbiol. 2024;9(6):1434–53. 10.1038/s41564-024-01705-x. 38834776 10.1038/s41564-024-01705-x PMC11153159

[32] Chung KM, Nang SC, Tang SS. The safety of bacteriophages in treatment of diseases caused by multidrug-resistant bacteria. Pharmaceuticals. 2023;16(10):1347. 10.3390/ph16101347. 37895818 10.3390/ph16101347 PMC10610463

[33] Abedon ST, García P, Mullany P, Aminov R, Editorial. Phage therapy: past, present and future. Front Microbiol. 2017;8:981. 10.3389/fmicb.2017.00981. 28663740 10.3389/fmicb.2017.00981 PMC5471325

[34] Ling H, Lou X, Luo Q, He Z, Sun M, Sun J. Recent advances in bacteriophage-based therapeutics: insight into the post-antibiotic era. Acta Pharm Sin B. 2022;12(12):4348–64. 10.1016/j.apsb.2022.05.007. 36561998 10.1016/j.apsb.2022.05.007 PMC9764073

[35] Sambrook J, Russell DW. Molecular cloning, a laboratory manual. 3rd ed. New York, NY, USA: Cold Spring Harbor Laboratory Press; 2001.

[36] Hietala V, Horsma-Heikkinen J, Carron A, Skurnik M, Kiljunen S. The removal of endo- and enterotoxins from bacteriophage preparations. Front Microbiol. 2019;10:1674. 10.3389/fmicb.2019.01674. 31396188 10.3389/fmicb.2019.01674 PMC6664067

[37] Schneider CA, Rasband WS, Eliceiri KW. NIH image to imageJ: 25 years of image analysis. Nat Methods. 2012;9:671–5. 22930834 10.1038/nmeth.2089 PMC5554542

[38] Kallio MA, Tuimala JT, Hupponen T, Klemelä P, Gentile M, Scheinin I, Koski M, Kärki J, Korpelainen EI. Chipster: user-friendly analysis software for microarray and other high-throughput data. BMC Genomics. 2011;12:507. 10.1186/1471-2164-12-507. 21999641 10.1186/1471-2164-12-507 PMC3215701

[39] Coil D, Jospin G, Darling AE. A5-miseq: an updated pipeline to assemble microbial genomes from illumina miseq data. Bioinformatics. 2015;31(4):587–9. 10.1093/bioinformatics/btu661. 25338718 10.1093/bioinformatics/btu661

[40] Garneau JR, Depardieu F, Fortier LC, Bikard D, Monot M. PhageTerm: a tool for fast and accurate determination of phage termini and packaging mechanism using next-generation sequencing data. Sci Rep. 2017;7:8292. 10.1038/s41598-017-07910-5. 28811656 10.1038/s41598-017-07910-5 PMC5557969

[41] Seemann T. Prokka: rapid prokaryotic genome annotation. Bioinformatics. 2014;30:2068–9. 10.1093/bioinformatics/btu153. 24642063 10.1093/bioinformatics/btu153

[42] Altschul SF, Madden TL, Schäffer AA, Zhang Z, Miller W, Lipman DJ. Gapped BLAST and PSI-BLAST: a new generation of protein database search programs. Nucleic Acids Res. 1997;25:3389–402. 10.1093/nar/25.17.3389. 9254694 10.1093/nar/25.17.3389 PMC146917

[43] Zimmermann L, Stephens A, Nam S-Z, Rau D, Kübler J, Lozajic M, Gabler F, Söding J, Lupas AN, Alva V. A completely reimplemented MPI bioinformatics toolkit with a new HHpred server at its core. J Mol Biol. 2018;430:2237–43. 10.1016/j.jmb.2017.12.007. 29258817 10.1016/j.jmb.2017.12.007

[44] Chan PP, Lin BY, Mak AJ, Lowe TM. tRNAscan-SE 2.0: improved detection and functional classification of transfer RNA genes. Nucleic Acids Res. 2021;49:9077–96. 10.1093/nar/gkab688. 34417604 10.1093/nar/gkab688 PMC8450103

[45] Laslett D, Canback B. ARAGORN, a program to detect tRNA genes and TmRNA genes in nucleotide sequences. Nucleic Acids Res. 2004;32:11–6. 10.1093/nar/gkh152. 14704338 10.1093/nar/gkh152 PMC373265

[46] Joensen KG, Scheutz F, Lund O, Hasman H, Kaas RS, Nielsen EM, Aarestrup FM. Real-time whole-genome sequencing for routine typing, surveillance, and outbreak detection of verotoxigenic *Escherichia coli*. J Clin Microbiol. 2014;52:1501–10. 10.1128/JCM.03617-13. 24574290 10.1128/JCM.03617-13 PMC3993690

[47] Bortolaia V, Kaas RS, Ruppe E, Roberts MC, Schwarz S, Cattoir V, Philippon A, Allesoe RL, Rebelo AR, Florensa AF, et al. ResFinder 4.0 for predictions of phenotypes from genotypes. J Antimicrob Chemother. 2020;75:3491–500. 10.1093/jac/dkaa345. 32780112 10.1093/jac/dkaa345 PMC7662176

[48] Meier-Kolthoff JP, Göker M. Genome-based phylogeny and classification of prokaryotic viruses. Bioinformatics. 2017;33:3396–404. 10.1093/bioinformatics/btx440. 29036289 10.1093/bioinformatics/btx440 PMC5860169

[49] Meier-Kolthoff JP, Auch AF, Klenk H-P, Göker M. Genome sequence-based species delimitation with confidence intervals and improved distance functions. BMC Bioinformatics. 2013;14:60. 10.1186/1471-2105-14-60. 23432962 10.1186/1471-2105-14-60 PMC3665452

[50] Lefort V, Desper R, Gascuel O. FastME 2.0: A comprehensive, accurate, and fast distance-based phylogeny inference program. Mol Biol Evol. 2015;32:2798–800. 10.1093/molbev/msv150. 26130081 10.1093/molbev/msv150 PMC4576710

[51] Farris JS. Estimating phylogenetic trees from distance matrices. Am Nat. 1972;106:645–67.

[52] Yu G. Using Ggtree to visualize data on tree-like structures. Curr Protoc Bioinforma. 2020;69:1–18. 10.1002/cpbi.96. 10.1002/cpbi.96 32162851

[53] Göker M, García-Blázquez G, Voglmayr H, Tellería MT, Martín MP. Molecular taxonomy of phytopathogenic fungi: A case study in *Peronospora*. PLoS ONE. 2009;4:8–10. 10.1371/journal.pone.0006319. 10.1371/journal.pone.0006319 PMC2712678 19641601

[54] Meier-Kolthoff JP, Hahnke RL, Petersen J, Scheuner C, Michael V, Fiebig A, Rohde C, Rohde M, Fartmann B, Goodwin LA, et al. Complete genome sequence of DSM 30083T, the type strain (U5/41T) of *Escherichia coli*, and a proposal for delineating subspecies in microbial taxonomy. Stand Genomic Sci. 2014;9:2. 10.1186/1944-3277-9-2. 25780495 10.1186/1944-3277-9-2 PMC4334874

[55] Moraru C, Varsani A, Kropinski AM. VIRIDIC– a novel tool to calculate the intergenomic similarities of prokaryote-infecting viruses. Viruses 2020;12. 10.3390/v12111268 10.3390/v12111268 PMC7694805 33172115

[56] Galaxy Community. The galaxy platform for accessible, reproducible, and collaborative data analyses: 2024 update. Nucleic Acids Res. 2024;52(W1):W83–94. 10.1093/nar/gkae410. 38769056 10.1093/nar/gkae410 PMC11223835

[57] Nishimura Y, Yamada K, Okazaki Y, Ogata H. DiGAlign: versatile and interactive visualization of sequence alignment for comparative genomics. Microbes Environ. 2024;39(1):ME23061. 10.1264/jsme2.ME23061. 38508742 10.1264/jsme2.ME23061 PMC10982109

[58] Patpatia S, Schaedig E, Dirks A, Paasonen L, Skurnik M, Kiljunen S. Rapid hydrogel-based phage susceptibility test for pathogenic bacteria. Front Cell Infect Microbiol. 2022;12. 10.3389/fcimb.2022.1032052. 10.3389/fcimb.2022.1032052 PMC9771388 36569196

[59] Lavigne R, Darius P, Summer EJ, Seto D, Mahadevan P, Nilsson AS, Ackermann HW, Kropinski AM. Classification of *Myoviridae* bacteriophages using protein sequence similarity. BMC Microbiol. 2009;9:224. 10.1186/1471-2180-9-224 19857251 10.1186/1471-2180-9-224 PMC2771037

[60] Krylov VN, Zhazykov Izh. Bakteriofag PhiKZ *Pseudomonas–v*ozmozhnaia model’ Dlia Izucheniia Geneticheskogo kontrolia morfogeneza [*Pseudomonas* bacteriophage PhiKZ–possible model for studying the genetic control of morphogenesis]. Genetika. 1978;14(4):678–85. 95987

[61] Latz S, Krüttgen A, Häfner H, Buhl EM, Ritter K, Horz HP. Differential effect of newly isolated phages belonging to PB1-Like, phiKZ-like and LUZ24-like viruses against multi-drug resistant *Pseudomonas aeruginosa* under varying growth conditions. Viruses. 2017;9(11):315. 10.3390/v9110315. 29077053 10.3390/v9110315 PMC5707522

[62] Virus T. 2022 Release. International Committee on Taxonomy of Viruses (ICTV). https://ictv.global/taxonomy. Accessed 26. February 2024.

[63] Francke C, Groot Kormelink T, Hagemeijer Y, Overmars L, Sluijter V, Moezelaar R, Siezen RJ. Comparative analyses imply that the enigmatic Sigma factor 54 is a central controller of the bacterial exterior. BMC Genomics. 2011;12:385. 10.1186/1471-2164-12-385. 21806785 10.1186/1471-2164-12-385 PMC3162934

[64] Liu X, Ye Y, Zhu Y, Wang L, Yuan L, Zhu J, Sun A. Involvement of RpoN in regulating motility, biofilm, resistance, and spoilage potential of *Pseudomonas fluorescens*. Front Microbiol. 2021;12:641844. 10.3389/fmicb.2021.641844. 34135871 10.3389/fmicb.2021.641844 PMC8202526

[65] Totten PA, Lara JC, Lory S. The RpoN gene product of *Pseudomonas aeruginosa* is required for expression of diverse genes, including the Flagellin gene. J Bacteriol. 1990;172(1):389–96. 10.1128/jb.172.1.389-396.1990 2152909 10.1128/jb.172.1.389-396.1990 PMC208444

[66] Chaikeeratisak V, Birkholz EA, Pogliano J. The phage nucleus and PhuZ spindle: defining features of the subcellular organization and speciation of nucleus-forming Jumbo phages. Front Microbiol. 2021;12:641317. 10.3389/fmicb.2021.641317. 34326818 10.3389/fmicb.2021.641317 PMC8314001

[67] Li Y, Guan J, Hareendranath S, Crawford E, Agard DA, Makarova KS, Koonin EV, Bondy-Denomy J. A family of novel immune systems targets early infection of nucleus-forming Jumbo phages. BioRxiv Preprint. 2022. 10.1101/2022.09.17.508391. 09.17.508391.

[68] Danis-Wlodarczyk K, Vandenheuvel D, Jang HB, Briers Y, Olszak T, Arabski M, Wasik S, Drabik M, Higgins G, Tyrrell J, et al. A proposed integrated approach for the preclinical evaluation of phage therapy in *Pseudomonas* infections. Sci Rep. 2016;6:28115. 10.1038/srep28115. 27301427 10.1038/srep28115 PMC4908380

[69] Chan BK, Sistrom M, Wertz JE, Kortright KE, Narayan D, Turner PE. Phage selection restores antibiotic sensitivity in MDR *Pseudomonas aeruginosa*. Sci Rep. 2016;6:26717. 10.1038/srep26717. 27225966 10.1038/srep26717 PMC4880932

[70] Esteves NC, Scharf BE. Flagellotropic bacteriophages: opportunities and challenges for antimicrobial applications. Int J Mol Sci. 2022;23(13):7084. 10.3390/ijms23137084. 35806089 10.3390/ijms23137084 PMC9266447

[71] Raimondo LM, Lundh NP, Martinez RJ. Primary adsorption site of phage PBS1: the flagellum of *Bacillus subtilis*. J Virol. 1968;2(3):256–64. 10.1128/JVI.2.3.256-264.1968. 4986906 10.1128/jvi.2.3.256-264.1968 PMC375608

[72] Jollick JD, Wright BL. A flagella specific bacteriophage for *Caulobacter*. J Gen Virol. 1974;22(2):197–205. 10.1099/0022-1317-22-2-197. 4820673 10.1099/0022-1317-22-2-197

[73] Guerrero-Ferreira RC, Viollier PH, Ely B, Poindexter JS, Georgieva M, Jensen GJ, Wright ER. Alternative mechanism for bacteriophage adsorption to the motile bacterium *Caulobacter crescentus*. PNAS USA. 2011;108(24):9963–8. 10.1073/pnas.1012388108. 21613567 10.1073/pnas.1012388108 PMC3116389

[74] Geiben-Lynn R, Sauber K, Lutz F. Flagellin inhibits *Myoviridae* phage PhiCTX infection of *Pseudomonas aeruginosa* strain GuA18: purification and mapping of binding site. Arch Microbiol. 2001;176(5):339–46. 10.1007/s002030100332 11702075 10.1007/s002030100332

[75] Ranta K, Skurnik M, Kiljunen S. fENko-Kae01 is a flagellum-specific Jumbo phage infecting *Klebsiella aerogenes*. BMC Microbiol. 2024;24(1):234. 10.1186/s12866-024-03387-1. 38951769 10.1186/s12866-024-03387-1 PMC11218385

[76] Jo D, Kim H, Lee Y, Kim J, Ryu S. Characterization and genomic study of EJP2, a novel Jumbo phage targeting antimicrobial resistant *Escherichia coli*. Front Microbiol. 2023;14:1194435. 10.3389/fmicb.2023.1194435. 37250060 10.3389/fmicb.2023.1194435 PMC10213699

[77] Olszak T, Danis-Wlodarczyk K, Arabski M, Gula G, Maciejewska B, Wasik S, Lood C, Higgins G, Harvey BJ, Lavigne R, Drulis-Kawa Z. *Pseudomonas aeruginosa* PA5oct Jumbo phage impacts planktonic and biofilm population and reduces its host virulence. Viruses. 2019;11(12):1089. 10.3390/v11121089. 31771160 10.3390/v11121089 PMC6950013

[78] Ouyang R, Costa AR, Cassidy CK, Otwinowska A, Williams VCJ, Latka A, Stansfeld PJ, Drulis-Kawa Z, Briers Y, Pelt DM, Brouns SJJ, Briegel A. High-resolution reconstruction of a Jumbo-bacteriophage infecting capsulated bacteria using hyperbranched tail fibers. Nat Commun. 2022;13(1):7241. 10.1038/s41467-022-34972-5. 36433970 10.1038/s41467-022-34972-5 PMC9700779

[79] Fields JL, Zhang H, Bellis NF, Petersen HA, Halder SK, Rich-New ST, Wu H, Wang F. Structural diversity and clustering of bacterial flagellar outer domains. Nat Commun. 2024;15:9500. 10.1038/s41467-024-53923-w. 39489766 10.1038/s41467-024-53923-w PMC11532411

[80] Pei R, Lamas-Samanamud GR. Inhibition of biofilm formation by T7 bacteriophages producing quorum-quenching enzymes. Appl Environ Microbiol. 2014;80(17):5340–8. 10.1128/AEM.01434-14. 24951790 10.1128/AEM.01434-14 PMC4136088

[81] Jiang Z, Zhang Y, Zhu X, Zhou Y, Qian Q, Gao X, Jiang Q, Zhang X. Involvement of RpoN in regulating stress response, biofilm formation and virulence of *Vibrio mimicus*. Aquaculture. 2024;578:740116. 10.1016/j.aquaculture.2023.740116.

[82] Chan BK, Turner PE, Kim S, Mojibian HR, Elefteriades JA, Narayan D. Phage treatment of an aortic graft infected with *Pseudomonas aeruginosa*. Evol Med Public Health. 2018;160–6. 10.1093/emph/eoy005. 10.1093/emph/eoy005 PMC5842392 29588855

[83] Erhardt M, Hughes KT. C-ring requirement in flagellar type III secretion is bypassed by FlhDC upregulation. Mol Microbiol. 2010;75:376– 93. 10.1111/j.1365-2958.2009.06973.x. 10.1111/j.1365-2958.2009.06973.x PMC3194100 19919668

[84] Xiao Y, Wigneshweraraj SR, Weinzierl R, Wang Y-P, Buck M. Construction and functional analyses of a comprehensive Σ54 site-directed mutant library using alanine–cysteine mutagenesis. Nucleic Acids Res. 2009;37(13):4482–97. 10.1093/nar/gkp419. 19474350 10.1093/nar/gkp419 PMC2715252

[85] Hendriksen JJ, Lee HJ, Bradshaw AJ, Namba K, Chevance FFV, Minamino T, Hughes KT. Genetic analysis of the *Salmonella* FliE protein that forms the base of the flagellar axial structure. mBio. 2021;12:e02392–21. 10.1128/mBio.02392-21. 34579566 10.1128/mBio.02392-21 PMC8546590

