# Supplementary material for: Isolation and characterization of fMGyn-Pae01, a phiKZ-like jumbo phage infecting Pseudomonas aeruginosa
Source: Virol J. 2025 Mar 4;22:55. doi: 10.1186/s12985-025-02679-w (PMC11877940; doi:10.1186/s12985-025-02679-w)
Supplement: Supplementary file 1 — Supplementary Material 1 [file 12985_2025_2679_MOESM1_ESM.docx]

**Table S1. Bacterial strains used in the study**

| **Bacterial species** | **Strain ID (S)** | **Origin, comments** | **fMGyn-Pae01 susceptibility, liquid method** |
| --- | --- | --- | --- |
| *Pseudomonas aeruginosa* | 6728 | Original host, HUS Diagnostic Center | + |
| *Pseudomonas aeruginosa* | 5513 | Human blood culture isolate, HUS Diagnostic Center | - |
| *Pseudomonas aeruginosa* | 5514 | Human blood culture isolate, HUS Diagnostic Center | +/- |
| *Pseudomonas aeruginosa* | 5525 | Human blood culture isolate, HUS Diagnostic Center | - |
| *Pseudomonas aeruginosa* | 5537 | Human faeces isolate, HUS Diagnostic Center | - |
| *Pseudomonas aeruginosa* | 5538 | Human urine isolate, HUS Diagnostic Center | - |
| *Pseudomonas aeruginosa* | 5539 | Human urine isolate, HUS Diagnostic Center | - |
| *Pseudomonas aeruginosa* | 5548 | Human faeces isolate, HUS Diagnostic Center | - |
| *Pseudomonas aeruginosa* | 5550 | Human blood culture isolate, HUS Diagnostic Center | - |
| *Pseudomonas aeruginosa* | 5551 | Human blood culture isolate, HUS Diagnostic Center | - |
| *Pseudomonas aeruginosa* | 5553 | Human blood culture isolate, HUS Diagnostic Center | - |
| *Pseudomonas aeruginosa* | 5668 | Clinical isolate, HUS Diagnostic Center | + |
| *Pseudomonas aeruginosa* | 5669 | Clinical isolate, HUS Diagnostic Center | +/- |
| *Pseudomonas aeruginosa* | 5670 | Clinical isolate, HUS Diagnostic Center | + |
| *Pseudomonas aeruginosa* | 5671 | Clinical isolate, HUS Diagnostic Center | + |
| *Pseudomonas aeruginosa* | 5672 | Clinical isolate, HUS Diagnostic Center | - |
| *Pseudomonas aeruginosa* | 5740 | Clinical isolate, HUS Diagnostic Center | + |
| *Pseudomonas aeruginosa* | 5741 | Clinical isolate, HUS Diagnostic Center | + |
| *Pseudomonas aeruginosa* | 5742 | Clinical isolate, HUS Diagnostic Center | - |
| *Pseudomonas aeruginosa* | 5743 | Clinical isolate, HUS Diagnostic Center | - |
| *Pseudomonas aeruginosa* | 5745 | Clinical isolate, HUS Diagnostic Center | +/- |
| *Pseudomonas aeruginosa* | 5746 | Clinical isolate, HUS Diagnostic Center | - |
| *Pseudomonas aeruginosa* | 5747 | Clinical isolate, HUS Diagnostic Center | - |
| *Pseudomonas aeruginosa* | 5826 | Clinical isolate, HUS Diagnostic Center | + |
| *Pseudomonas aeruginosa* | 5827 | Clinical isolate, HUS Diagnostic Center | + |
| *Pseudomonas aeruginosa* | 5828 | Clinical isolate, HUS Diagnostic Center | + |
| *Pseudomonas aeruginosa* | 5829 | Clinical isolate, HUS Diagnostic Center | + |
| *Pseudomonas aeruginosa* | 5831 | Clinical isolate, HUS Diagnostic Center | + |
| *Pseudomonas aeruginosa* | 5832 | Clinical isolate, HUS Diagnostic Center | + |
| *Pseudomonas aeruginosa* | 5833 | Clinical isolate, HUS Diagnostic Center | + |
| *Pseudomonas aeruginosa* | 5834 | Clinical isolate, HUS Diagnostic Center | + |
| *Pseudomonas aeruginosa* | 5835 | Clinical isolate, HUS Diagnostic Center | + |
| *Pseudomonas aeruginosa* | 5836 | Clinical isolate, HUS Diagnostic Center | + |
| *Pseudomonas aeruginosa* | 5837 | Clinical isolate, HUS Diagnostic Center | - |
| *Pseudomonas aeruginosa* | 5838 | Clinical isolate, HUS Diagnostic Center | - |
| *Pseudomonas aeruginosa* | 5839 | Clinical isolate, HUS Diagnostic Center | - |
| *Pseudomonas aeruginosa* | 5840 | Clinical isolate, HUS Diagnostic Center | + |
| *Pseudomonas aeruginosa* | 5841 | Clinical isolate, HUS Diagnostic Center | + |
| *Pseudomonas aeruginosa* | 5842 | Clinical isolate, HUS Diagnostic Center | - |
| *Pseudomonas aeruginosa* | 5844 | Clinical isolate, HUS Diagnostic Center | +/- |
| *Pseudomonas aeruginosa* | 5845 | Clinical isolate, HUS Diagnostic Center | - |
| *Pseudomonas aeruginosa* | 5846 | Clinical isolate, HUS Diagnostic Center | + |
| *Pseudomonas aeruginosa* | 5847 | Clinical isolate, HUS Diagnostic Center | + |
| *Pseudomonas aeruginosa* | 5848 | Clinical isolate, HUS Diagnostic Center | +/- |
| *Pseudomonas aeruginosa* | 6327 | University of Jyväskylä, Matti Jalasvuori | + |
| *Pseudomonas aeruginosa* | 6329 | University of Jyväskylä, Matti Jalasvuori | + |
| *Pseudomonas aeruginosa* | 6331 | University of Jyväskylä, Matti Jalasvuori | - |
| *Pseudomonas aeruginosa* | 6663 | Clinical isolate, HUS Diagnostic Center | - |
| *Pseudomonas aeruginosa* | 6665 | Clinical isolate, HUS Diagnostic Center | - |
| *Pseudomonas aeruginosa* | 6666 | Clinical isolate, HUS Diagnostic Center | - |
| *Pseudomonas aeruginosa* | 6667 | Clinical isolate, HUS Diagnostic Center | - |
| *Pseudomonas aeruginosa* | 6668 | Clinical isolate, HUS Diagnostic Center | - |
| *Pseudomonas aeruginosa* | 6671 | Clinical isolate, HUS Diagnostic Center | - |
| *Pseudomonas aeruginosa* | 6672 | Clinical isolate, HUS Diagnostic Center | - |
| *Pseudomonas aeruginosa* | 6673 | Clinical isolate, HUS Diagnostic Center | - |
| *Pseudomonas aeruginosa* | 6674 | Clinical isolate, HUS Diagnostic Center | + |
| *Pseudomonas aeruginosa* | 6675 | Clinical isolate, HUS Diagnostic Center | + |
| *Pseudomonas aeruginosa* | 6676 | Clinical isolate, HUS Diagnostic Center | + |
| *Pseudomonas aeruginosa* | 6677 | Clinical isolate, HUS Diagnostic Center | + |
| *Pseudomonas aeruginosa* | 6678 | Clinical isolate, HUS Diagnostic Center | - |
| *Pseudomonas aeruginosa* | 6679 | Clinical isolate, HUS Diagnostic Center | - |
| *Pseudomonas aeruginosa* | 6680 | Clinical isolate, HUS Diagnostic Center | - |
| *Pseudomonas aeruginosa* | 6681 | Clinical isolate, HUS Diagnostic Center | - |
| *Pseudomonas aeruginosa* | 6682 | Clinical isolate, HUS Diagnostic Center | - |
| *Pseudomonas aeruginosa* | 6704 | IATSO1 [1, 2] | + |
| *Pseudomonas aeruginosa* | 6705 | IATSO2 [1, 2] | - |
| *Pseudomonas aeruginosa* | 6706 | IATSO3 [1, 2] | - |
| *Pseudomonas aeruginosa* | 6707 | IATSO4 [1, 2] | - |
| *Pseudomonas aeruginosa* | 6708 | IATSO5, PAO1 [1, 2] | + |
| *Pseudomonas aeruginosa* | 6709 | IATSO6 [1, 2] | + |
| *Pseudomonas aeruginosa* | 6710 | IATSO7 [1, 2] | + |
| *Pseudomonas aeruginosa* | 6711 | IATSO8 [1, 2] | - |
| *Pseudomonas aeruginosa* | 6712 | IATSO9 [1, 2] | + |
| *Pseudomonas aeruginosa* | 6713 | IATSO10, PA14 [1, 2] | -/+ |
| *Pseudomonas aeruginosa* | 6714 | IATSO11 [1, 2] | + |
| *Pseudomonas aeruginosa* | 6715 | IATSO12, PA7 [1, 2] | - |
| *Pseudomonas aeruginosa* | 6716 | IATSO13 [1, 2] | - |
| *Pseudomonas aeruginosa* | 6717 | IATSO14 [1, 2] | - |
| *Pseudomonas aeruginosa* | 6718 | IATSO15 [1, 2] | - |
| *Pseudomonas aeruginosa* | 6719 | IATSO16 [1, 2] | - |
| *Pseudomonas aeruginosa* | 6720 | IATSO17 [1, 2] | + |
| *Pseudomonas aeruginosa* | 6721 | IATSO18 [2, 3] | - |
| *Pseudomonas aeruginosa* | 6722 | IATSO19 [2, 3] | - |
| *Pseudomonas aeruginosa* | 6723 | IATSO20 [2, 3] | + |
| *Pseudomonas aeruginosa* | 6724 | *DrmlC*; LPS phenotype: OSA- (O-specific antigen) CPA- (common polysaccharide antigen), truncated core [4] | - |
| *Pseudomonas aeruginosa* | 6725 | ΔwbpM; LPS phenotype: OSA- CPA+ [5] | + |
| *Pseudomonas aeruginosa* | 6726 | *Dpa5456;* LPS phenotype: OSA+ CPA- [6] | + |
| *Pseudomonas aeruginosa* | 6727 | *ΔwbpL*.; LPS phenotype: OSA- CPA- [7] | - |
| *Pseudomonas aeruginosa* | 6732 | Clinical isolate, HUS Diagnostic Center | + |
| *Pseudomonas aeruginosa* | 6734 | Clinical isolate, HUS Diagnostic Center | + |
| *Pseudomonas aeruginosa* | 6827 | Clinical isolate, Germany | + |
| *Pseudomonas aeruginosa* | 262 | PA012/RP1 | + |
| *Pseudomonas aeruginosa* | 5667 | Clinical isolate, HUS Diagnostic Center | +/- |
| *Pseudomonas aeruginosa* | 5739 | Clinical isolate, HUS Diagnostic Center | - |
| *Pseudomonas aeruginosa* | 5744 | Clinical isolate, HUS Diagnostic Center | - |
| *Pseudomonas aeruginosa* | 6328 | University of Jyväskylä, Matti Jalasvuori | + |
| *Pseudomonas aeruginosa* | 6330 | University of Jyväskylä, Matti Jalasvuori | + |
| *Pseudomonas aeruginosa* | 6332 | University of Jyväskylä, Matti Jalasvuori | - |
| *Pseudomonas aeruginosa* | 6333 | University of Jyväskylä, Matti Jalasvuori | - |
| *Pseudomonas aeruginosa* | 6458 | Clinical isolate, HUS Diagnostic Center | - |
| *Pseudomonas aeruginosa* | 6459 | Clinical isolate, HUS Diagnostic Center | + |
| *Pseudomonas aeruginosa* | 6558 | Clinical wound infection isolate | + |
| *Pseudomonas putida* | 255 | AC59 | - |
| *Pseudomonas putida* | 256 | PAW15 (TOL) | - |
| *Pseudomonas putida* | 257 | PAW1 (TOL) | - |
| *Pseudomonas putida* | 5843 | Clinical isolate, HUS Diagnostic Center | - |
| *Escherichia coli* | 6741 | Human urine isolate, HUS Diagnostic Center | - |
| *Escherichia coli* | 6742 | Human blood culture isolate, HUS Diagnostic Center | - |
| *Escherichia coli* | 6729 | Clinical isolate, HUS Diagnostic Center | - |
| *Escherichia coli* | 6730 | Clinical isolate, HUS Diagnostic Center | - |
| *Escherichia coli* | 6731 | Clinical isolate, HUS Diagnostic Center | - |
| *Escherichia coli* | 6498 | Clinical isolate, HUS Diagnostic Center | - |
| *Escherichia coli* | 6501 | Clinical isolate, HUS Diagnostic Center | - |
| *Escherichia coli* | 6503 | Clinical isolate, HUS Diagnostic Center | - |
| *Escherichia coli* | 6508 | Clinical isolate, HUS Diagnostic Center | - |
| *Escherichia coli* | 6509 | Clinical isolate, HUS Diagnostic Center | - |
| *Klebsiella pneumoniae* | 6815 | Human nasal isolate, University Hospital of Wurzberg | - |
| *Klebsiella pneumoniae* | 6740 | Clinical isolate, HUS Diagnostic Center | - |
| *Klebsiella pneumoniae* | 6738 | Clinical isolate, HUS Diagnostic Center | - |
| *Klebsiella pneumoniae* | 6596 | Clinical isolate, HUS Diagnostic Center | - |
| *Klebsiella pneumoniae* | 6470 | Clinical isolate, HUS Diagnostic Center | - |
| *Klebsiella pneumoniae* | 6322 | University of Jyväskylä, Matti Jalasvuori | - |
| *Klebsiella pneumoniae* | 6323 | University of Jyväskylä, Matti Jalasvuori | - |
| *Klebsiella pneumoniae* | 6324 | University of Jyväskylä, Matti Jalasvuori | - |
| *Klebsiella pneumoniae* | 6325 | University of Jyväskylä, Matti Jalasvuori | - |
| *Klebsiella pneumoniae* | 6326 | University of Jyväskylä, Matti Jalasvuori | - |
| *Salmonella typhimurium* | 1356 | TV-163 [8] | - |
| *Salmonella typhimurium* | 1357 | [9] | - |
| *Salmonella typhimurium* | 1422 | ATCC13311 | - |
| *Salmonella typhimurium* | 1423 | Clinical sample, department of medical microbiology, University of Turku | - |
| *Salmonella typhimurium* | 1426 | Laboratory strain, University of Helsinki | - |
| *Salmonella typhimurium* | 1427 | Laboratory strain, University of Helsinki | - |
| *Salmonella typhimurium* | 1860 | Laboratory strain, University of Helsinki | - |
| *Salmonella typhimurium* | 2547 | Laboratory strain, University of Helsinki | - |
| *Salmonella typhimurium* | 6474 | Clinical sample, department of medical microbiology, University of Turku | - |
| *Salmonella typhimurium* | 6670 | Laboratory strain, University of Helsinki | - |
| *Proteus mirabilis* | 5002 | O:27 (50/57) | - |
| *Proteus mirabilis* | 5003 | O:28 (51/57) | - |
| *Proteus mirabilis* | 5004 | O:3 (S1959) | - |
| *Proteus mirabilis* | 5993 | O:27 (50/57/pIP::cat) | - |
| *Proteus mirabilis* | 6461 | Clinical isolate, HUS Diagnostic Center | - |
| *Proteus mirabilis* | 6463 | Clinical isolate, HUS Diagnostic Center | - |
| *Proteus mirabilis* | 6473 | Clinical isolate, HUS Diagnostic Center | - |

+ infected efficiently, +/- infected with low efficiency, - did not infect

**Table S2. The strain names and Sequence Read Archive (SRA) ID and accession numbers of the *Pseudomonas aeruginosa* host strain and the fMGyn-Pae01 resistant mutants.**

| **Strain** | **Strain ID in SRA database** | **Accession number** |
| --- | --- | --- |
| S6728 | Pa_6728 | SAMN40738801 |
| S6728-M5 | Pa_7284 | SAMN40738802 |
| S6728-M7 | Pa_7285 | SAMN40738803 |
| S6728-M8 | Pa_7286 | SAMN40738804 |
| S6728-M9 | Pa_7287 | SAMN40738805 |
| S6728-M10 | Pa_7288 | SAMN40738806 |

**Table S3. Comparison of Chimallin core genome between phiKZ and fMGyn-Pae01.** BLASTP comparisons of 20 ORFs belonging to Chimalliviridae core genome and being present in only chimallin-containing phages and phiKZ as suggested by Prichard et al. [10].

| **Core Genome Number** | **Putative Function** | **phiKZ** | **fMGyn-Pae01** | **Coverage / Identity to phiKZ homolog** |
| --- | --- | --- | --- | --- |
| cg1 | hypothetical protein | gp049, NP_803615.1 | WNV47731.1 | 100 % / 100 % |
| cg3 | hypothetical protein  (structural head protein) | gp052, NP_803618.2 | WNV47735.1 | 95 % / 99.71 % |
| cg4 | Nuclear Shell Protein (chimallin) | gp054, NP_803620.1 | WNV47737.1 | 100 % / 99.84 % |
| cg6 | hypothetical protein | gp059, NP_803625.1 | WNV47745.1 | 100 % / 99.51 % |
| gc7 | hypothetical protein | gp062, NP_803628.1 | WNV47748.1 | 100 % / 99.26 % |
| cg9 | hypothetical protein | gp066, NP_803632.1 | WNV47752.1 | 100 % / 99.64 % |
| cg12 | hypothetical protein | gp069, NP_803635.1 | WNV47755.1 | 100 % / 99.46 % |
| cg19 | hypothetical protein | gp079, NP_803645.2 | WNV47771.1 | 100 % / 99.65 % |
| cg26 | putative virion structural protein | gp090, NP_803656.1 | WNV47782.1 | 100 % / 100 % |
| cg27 | virion structural protein/internal head | gp093, NP_803659.1,  gp162 NP_803728.1  gp163 NP_803729.1 | WNV47785.1  WNV47863.1  WNV47864.1 | 100 % / 100 %  100 % / 100 %  100 % / 99.75 % |
| cg30 | hypothetical protein | gp100, NP_803666.1 | WNV47792.1 | 100 % / 100 % |
| cg35 | hypothetical protein | gp122, NP_803688.1 | WNV47821.1 | 100 % / 100 % |
| cg41 | virion structural protein | gp130, NP_803696.1 | WNV47829.1 | 100 % / 99.77 % |
| cg45 | hypothetical protein | gp161, NP_803727.2 | WNV47862.1 | 96 % / 100 % |
| cg46 | virion structural protein | gp157, NP_803723.1 | WNV47858.1 | 100 % / 100 % |
| cg48 | hypothetical protein (structural head protein) | gp153 NP_803719.1 | WNV47853.1 | 100 % / 100 % |
| cg51 | hypothetical protein | gp147, NP_803713.1 | WNV47847.1 | 100 % / 99.16 % |
| cg52 | hypothetical protein | gp171, NP_803737.1 | WNV47875.1 | 100 % / 100 % |
| cg58 | hypothetical protein  (structural head protein) | gp177, NP_803743.1 | WNV47881.1 | 100 % / 100 % |
| cg65 | putative structural protein | gp026, NP_803592.1 | WNV47701.1 | 100 % / 100 % |

**Table S4. Results of motility assay**

| **Strain** | **Colony diameter (cm)** | **SD** | **P-value** |
| --- | --- | --- | --- |
| S6728 | 5.38 | 1.25 |  |
| S6728-M5 | 4.24 | 1.00 | 0.004 |
| S6728-M7 | 3.72 | 0.87 | 0.019 |
| S6728-M8 | 3.09 | 1.30 | 0.000010 |
| S6728-M9 | 2.64 | 0.66 | 0.00012 |
| S6728-M10 | 2.74 | 0.49 | 0.00013 |

**References**

1. Liu PV, Matsumoto H, Kusama H, Bergan TOM. Survey of heat-stable, major somatic antigens of *Pseudomonas aeruginosa.* Int J Sys. Bacteriol 1983;33:256–64. https://doi.org/10.1099/00207713-33-2-256
2. Lam JS, Taylor V., Islam ST, Hao Y, Kocíncová D. Genetic and Functional Diversity of *Pseudomonas aeruginosa* Lipopolysaccharide. Front Microbiol 2011;2:118. https://doi.org/10.3389/fmicb.2011.00118
3. Liu PV, Wang S. Three new major somatic antigens of *Pseudomonas aeruginosa*. J Clin Microbiol 1990;28(5):922–5. https://doi.org/10.1128/jcm.28.5.922-925.1990
4. Rahim R, Burrows LL, Monteiro MA, Perry MB, Lam JS. Involvement of the rml locus in core oligosaccharide and O polysaccharide assembly in *Pseudomonas aeruginosa*. Microbiology (Reading, England) 2000;146(11):2803–14. https://doi.org/10.1099/00221287-146-11-2803
5. Creuzenet C, Lam JS. Topological and functional characterization of WbpM, an inner membrane UDP-GlcNAc C6 dehydratase essential for lipopolysaccharide biosynthesis in *Pseudomonas aeruginosa*. Mol Microbiol 2001;41(6):1295–310. https://doi.org/10.1046/j.1365-2958.2001.02589.x
6. Hao Y, King JD, Huszczynski S, Kocíncová D, Lam JS. Five new genes are important for common polysaccharide antigen biosynthesis in *Pseudomonas aeruginosa*. mBio 2013;4(1):e00631-12. https://doi.org/10.1128/mBio.00631-12
7. Rocchetta HL, Burrows LL, Pacan JC, Lam JS. Three rhamnosyltransferases responsible for assembly of the A-band D-rhamnan polysaccharide in *Pseudomonas aeruginosa*: a fourth transferase, WbpL, is required for the initiation of both A-band and B-band lipopolysaccharide synthesis. Mol Microbiol 1998;28(6):1103–19. https://doi.org/10.1046/j.1365-2958.1998.00871.x
8. Subbaiah TV, Stocker BA. Rough Mutants of *Salmonella Typhimurium.* I. Genetics. Nature 1964;201:1298–9. https://doi.org/10.1038/2011298a0
9. Nikaido H, Levinthal M, Nikaido K, Nakane K. Extended deletions in the histidine-rough-B region of the *Salmonella* chromosome. Proc Natl Acad Sci U S A 1967;57(6):1825–32. https://doi.org/10.1073/pnas.57.6.1825
10. Prichard A, Lee J, Laughlin TG, Lee A, Thomas KP, Sy AE, Spencer T, Asavavimol A, Cafferata A, Cameron M, et al. Identifying the core genome of the nucleus-forming bacteriophage family and characterization of Erwinia phage RAY. Cell Rep 2023;42(5):112432. https://doi.org/10.1016/j.celrep.2023.112432
